# Negative feedback regulation of karrikin signaling in *Arabidopsis thaliana* by an antagonistic paralog of karrikin receptors

**DOI:** 10.1073/pnas.2525145123

**Published:** 2026-09-08

**Authors:** Qingtian Li, Sun Hyun Chang, Andrew Tuckey, Claudia Sepulveda, Kartikye Varshney, Dan Li, Caroline Gutjahr, Mark T. Waters, David C. Nelson

**Affiliations:** ^a^ Department of Botany and Plant Sciences, University of California, Riverside, CA 92521; ^b^Yazhouwan National Laboratory, Sanya 572025, China; ^c^ School of Molecular Sciences, University of Western Australia, Perth, WA 6009, Australia; ^d^ Max-Planck-Institut für Molekulare Pflanzenphysiologie, Potsdam-Golm 14476, Germany

**Keywords:** karrikins, signaling, feedback, synthetic enhancement

## Abstract

Karrikins (KARs) are a class of butenolide molecules discovered in smoke hypothesized to mimic an undiscovered plant hormone, KAI2 ligand (KL). KAR/KL signaling regulates germination, seedling development, stress tolerance, and symbiotic interactions with soil microbes, among other traits. KAR/KL signaling is initiated by KARRIKIN INSENSITIVE2 (KAI2), an ɑ/β-hydrolase related to the strigolactone enzyme-receptor DWARF14 (D14). Activated KAI2 forms protein–protein interactions that trigger proteasomal degradation of a transcriptional regulator, SUPPRESSOR OF MAX2 1 (SMAX1), initiating changes in gene expression. *D14-LIKE2* (*DLK2*), an ancient paralog of *KAI2* and *D14*, is a prominent transcriptional marker of KAR/KL signaling in many plants that has uncertain function. We find that DLK2 forms a negative feedback loop that attenuates KAR/KL signaling in *Arabidopsis thaliana*. This mechanism complements that of *KARRIKIN UPREGULATED F-BOX1* (*KUF1*), which putatively restricts KAR/KL metabolism through targeted protein degradation. Loss-of-function mutations of *DLK2* show little effect alone, but synthetically enhance the constitutive KAR/KL responses of *kuf1* seedlings. Overexpression of DLK2 proteins from several plants increases the abundance of a SMAX1 ratiometric reporter. DLK2 does not require nuclear localization to protect SMAX1, suggesting its function is independent of interactions with SMAX1 or its transcriptional regulator partners. DLK2 hydrolyzes a profluorescent, desmethyl butenolide reporter molecule that is putatively analogous to KL. We hypothesize that DLK2 catabolizes KAI2 ligand(s) without participating in KAR/KL signaling directly. This functional antagonism could have evolved after *KAI2* gene duplication through subfunctionalizing mutations that disrupted protein–protein interactions while preserving enzymatic activity.

## Significance

Plants use hormones—small, mobile, chemical or peptide signals—to regulate developmental and physiological responses to environmental and biotic cues. Karrikins (KARs) are chemicals found in smoke that affect the growth of many plants, but are not made by plants. Genetic studies of how plants sense and respond to KARs have led to the hypothesis that KARs mimic an undiscovered plant hormone, “KAI2 ligand” (KL). Determining the structure of KL and how it is made may unlock new tools to control plant growth and improve productivity. Here we identify a mechanism by which plants feedback-regulate KAR/KL signaling, potentially by limiting KL abundance. This feedback is accomplished by a sister protein of the KAR/KL receptor that evolved antagonistic functions.

Karrikins (KARs) are a class of butenolide molecules found in plant-derived smoke that are produced from pyrolysis of carbohydrates such as xylose (1–3). KARs were discovered as potent stimulants of seed germination for several species that emerge after fire (1). However, plant responses to KARs are not limited to fire-adapted species or to seed germination (3). For example, seed germination, seedling photomorphogenesis, and root hair development of *Arabidopsis thaliana* are enhanced by KAR treatment (4–6).

An ɑ/β-hydrolase, KARRIKIN INSENSITIVE2 (KAI2)/HYPOSENSITIVE TO LIGHT (HTL)/D14-LIKE (D14L), is required for KAR responses in plants (7–14). Many biochemical assays have shown that KAI2 protein can bind KAR_1_ in vitro, but typically with lower affinity than expected from bioactive concentrations (15–21). This genetic and biochemical evidence initially led to the conclusion that KAI2 is a KAR receptor (15). Contradicting this idea, several in vitro and *ex planta* assays have demonstrated that KAI2 is not activated by KARs outside of plants (19, 22–24). Therefore, KAI2 probably recognizes a metabolite(s) of KAR produced by plants, rather than KARs themselves. A bioactive KAR metabolite(s) has not been identified, however.

Loss of *KAI2* has many effects on plant growth, including enhanced seed dormancy, reduced seedling photomorphogenesis (i.e., longer hypocotyls, smaller cotyledons, reduced light-responsive gene expression, and hyponastic cotyledon/leaf orientation), reduced root hair density and root hair elongation, reduced survival under drought, and abolished or reduced symbiotic interactions with arbuscular mycorrhizal fungi (6–8, 10, 11, 13, 25, 26). Many *kai2* mutant phenotypes are opposite to the growth effects of KAR treatments (4–7). This suggests that plants lacking KAI2 cannot respond to an endogenous signal(s) that KARs or KAR metabolites mimic (27). This hypothetical signal(s) is commonly referred to as **“**KAI2 ligand” (KL) in the literature and is anticipated to be present throughout land plants (14, 22, 28, 29).

KAR/KL signaling involves an SCF-type (Skp1, Cullin, F-box) E3 ubiquitin ligase-dependent mechanism, similar to the plant hormones auxin, jasmonic acid, gibberellin, and strigolactone (14, 30). Upon activation, KAI2 associates with an F-box protein, MORE AXILLARY GROWTH2 (MAX2), and a transcriptional regulator, SUPPRESSOR OF MAX2 1 (SMAX1) or its paralog SMAX1-LIKE2 (SMXL2) (23, 31). The KAI2-SCF^MAX2^ complex polyubiquitinates SMAX1 and SMXL2 proteins, marking them for rapid degradation by the 26S proteasome (31) (Fig. 2). The destruction of SMAX1 and SMXL2 initiates downstream transcriptional responses through the direct relief of transcriptional repression and by affecting the DNA-binding ability, availability, or stability of transcriptional regulator proteins (31–34).

The mechanism of KAR/KL signaling is very similar to that of strigolactone (SL). An ancient paralog of *KAI2* known as *DWARF14* (*D14*)/*DECREASED APICAL DOMINANCE2 (DAD2)/RAMOSUS3* (*RMS3*) encodes an SL receptor in angiosperms. D14 hydrolyzes SL, albeit slowly (35–38). During SL binding and/or hydrolysis, D14 associates with MAX2 and a subset of SMAX1 homologs in the SMAX1-LIKE (SMXL) family (i.e., SMXL6, SMXL7, and SMXL8 in *Arabidopsis*, or DWARF53 (D53) in rice). These targets of D14 are degraded within a few minutes of SL treatment, triggering transcriptional changes and downstream growth responses such as reduced axillary branching or tillering (39–41). Under osmotic stress or when exogenous SL is applied, D14 can also target SMAX1 and SMXL2 for degradation in *Arabidopsis* (31, 42). However, *d14* does not show phenotypes associated with SMAX1/SMXL2 accumulation like *kai2* does (7). Therefore, regulation of SMAX1/SMXL2 by SL is not typical, at least for *Arabidopsis* seed and seedlings.

Feedback regulation that restricts the intensity or duration of a signal is commonly observed in biological signaling systems. One mechanism for negative feedback regulation of KAR/KL signaling is mediated by *KARRIKIN UPREGULATED F-BOX1* (*KUF1*), which is transcriptionally induced after SMAX1 and SMXL2 degradation (43). The phenotypes of an *Arabidopsis kuf1* loss-of-function mutant are consistent with increased KAR/KL signaling, including shortened hypocotyls, enlarged cotyledons, increased drought tolerance, and differential expression of several KAR/KL-regulated genes (43, 44). Epistasis analysis has shown that *kuf1* phenotypes are dependent on *KAI2* and *MAX2*, indicating that KUF1 acts upstream of KAI2-SCF^MAX2^ signaling (43). Combined with the observation that *KUF1* expression is positively regulated by KAI2-SCF^MAX2^ activity, this implies that *KUF1* forms a negative feedback loop. Unexpectedly, *kuf1* seedlings have hypersensitive responses to KAR_1_ but not to KAR_2_ nor to *rac*-GR24, a racemic mixture of synthetic KAI2 and D14 agonists. This suggests that KUF1 affects the ability of KAI2 to perceive specific molecules (i.e., KAR_1_), rather than increasing KAI2-SCF^MAX2^ signaling overall (43). KUF1 is hypothesized to restrict the biosynthesis of KL and the metabolism of KAR_1_ into a bioactive ligand of KAI2 (43). Upregulation of *KUF1* in response to KAR treatment, GR24 treatment, or loss of SMAX1 is observed in lettuce, pea, rice, *Brachypodium distachyon*, and *Physcomitrium patens*, suggesting this may be a broadly conserved regulatory mechanism for maintaining homeostasis of KAR/KL signaling (9, 13, 45, 46).

Another prominent response to KAR/KL signaling is increased expression of *D14-LIKE2* (*DLK2)*. *DLK2* expression is regulated by *SMAX1* and *SMXL2*, and not by *SMXL6*, *SMXL7*, and *SMXL8* (31, 33). Although D14 promotes *DLK2* expression after exogenous SL treatment via crosstalk with SMAX1/SMXL2, *DLK2* transcript abundance is normal in *d14* and only reduced in *kai2* and *max2* (7, 31, 33). Thus, we consider *DLK2* to be a transcriptional marker of KAR/KL signaling specifically, and not SL signaling.

*DLK2* is an ancient paralog of *D14* and *KAI2* whose function has long remained elusive. DLK2 proteins share a conserved Ser-His-Asp catalytic triad with D14 and KAI2, implying that DLK2 also has hydrolytic activity. The catalytic triad residues are essential for the enzymatic and signaling functions of D14 and KAI2, with the exception that a D218A substitution in D14 allows SL-binding to be sufficient to initiate signal transduction (22, 38). *Arabidopsis* DLK2 hydrolyzes a SL with an unnatural stereochemical configuration, (–)-5-deoxystrigol, in vitro but this activity is quite weak compared to D14, which is itself an enzyme with a slow SL turnover rate (35, 37, 47). The rice DLK2 ortholog D14L2a also shows weak hydrolytic activity toward *rac*-GR24 that seems qualitatively similar to petunia DAD2/D14 (48). DLK2 could be a poor enzyme, but it seems more likely that an appropriate substrate for DLK2 has not yet been identified. Notably, the catalytic efficiency of DLK2 against a generic hydrolase substrate, *para*-nitrophenyl acetate, is 625 times that of D14 and 6.7 times that of KAI2 (49).

Regardless of what substrate DLK2 hydrolyzes, it is unlikely to participate in an SCF^MAX2^-dependent signaling mechanism. KAI2 and D14 proteins share a highly conserved set of residues that form an interaction surface with MAX2 (36, 50, 51). Any one of the missense mutations G158E, P161D, E174A, R177A, or F180R severely decreases or abolishes D14 interactions with MAX2 (36, 52). Compared to D14, DLK2 naturally has the combined equivalent of G158D, P161S, E174R, R177K, and F180K substitutions. These nonconservative amino acid changes, some of which are very similar to characterized D14 mutations, strongly suggest that DLK2 cannot associate with MAX2 (47). Perhaps providing further support, DLK2 protein abundance is not affected by *rac*-GR24 treatment, while D14 is degraded in a mostly MAX2-dependent manner in the hours after its activation (47, 53, 54).

Despite *DLK2* having one of the most consistent and strongest transcriptional responses to KAR treatment or the loss of SMAX1 and SMXL2, *dlk2* mutants in *A. thaliana* have shown few phenotypic differences from wild type (WT). The *dlk2* mutant has a reduced requirement for light during seed germination (55). But, the hypocotyl length and cotyledon size of *dlk2* seedlings have been reported to be normal, and *dlk2* seedlings show WT responses to KAR_1_, KAR_2_, and the individual stereoisomer components of *rac*-GR24, GR24^5DS^ (a SL analog) and GR24*^ent^*^-5DS^ (a putative KAR/KL analog) (7, 47). Furthermore, the addition of a *dlk2* mutation does not change the growth of *kai2* or *d14* mutant seedlings, so the lack of a *dlk2* phenotype is not due to functional redundancy of *DLK2* with *KAI2* or *D14* (47). Shoot branching is also normal in *dlk2* mutants (7). *DLK2* overexpression, however, can affect *Arabidopsis* seedling growth. Some *DLK2* overexpression lines show elongated hypocotyls under low light. Curiously, *DLK2* overexpression has been reported to increase hypocotyl length and cotyledon size in *dlk2 d14 kai2* triple mutants, suggesting the possibility of a *D14*- and *KAI2*-independent effect on growth (47).

Exciting clues about *DLK2* function have emerged with the discovery that *DLK2* may influence symbiotic interactions between plant roots and arbuscular mycorrhizal fungi (AMF). *SlDLK2*, a tomato (*Solanum lycopersicum*) ortholog of *DLK2*, is upregulated in roots undergoing colonization by AMF. Moreover, *SlDLK2* expression occurs specifically in arbuscule-containing (AMF-colonized) cortex cells (56). A similar pattern of AMF-induced expression has been observed for *DLK2* orthologs in rice (*Oryza sativa*) and the grass *Brachypodium distachyon* (13, 48). Hairy root transformation of tomato with *SlDLK2* overexpression and silencing constructs has provided evidence that *SlDLK2* inhibits the overall amount of colonization by AMF as well as the growth and branching of fungal arbuscules. *SlDLK2* overexpression causes similar effects on root colonization in a legume, *Medicago truncatula*. Therefore, SlDLK2 acts in a negative feedback loop that restricts AMF colonization. Strikingly, 42% of the genes downregulated in nonmycorrhizal roots that overexpressed *SlDLK2* were upregulated in roots undergoing mycorrhization. Because these transcriptional effects of *SlDLK2* overexpression occurred in the absence of AMF, it implies that SlDLK2 does not require an AMF-derived or AMF-induced signal to act (56). Instead, SlDLK2 may regulate responses to an endogenous plant signal(s) that is relevant to mycorrhization. Indeed, *SlDLK2* overexpression reduces the abundance of jasmonic acid, abscisic acid, the SL solanacol, and possibly the auxin indole acetic acid in tomato roots; as all of these hormones positively contribute to mycorrhization, these changes may explain how DLK2 affects AM (57). Alternatively, considering that root colonization by AMF is regulated by KAI2-SCF^MAX2^ (8, 10, 11, 13) and that DLK2 is homologous to the KL receptor KAI2, it might be that DLK2 attenuates responses to KL.

In this study, we revisited the function of *DLK2* in *A. thaliana*. We find that DLK2 has a role in negative feedback regulation of KAR/KL signaling, putatively through catabolism of KL, that is complementary to the proposed role of KUF1 in attenuating KAR/KL metabolism.

## Results

### Loss of *DLK2* Synthetically Enhances *kuf1*.

**Fig. 1.**
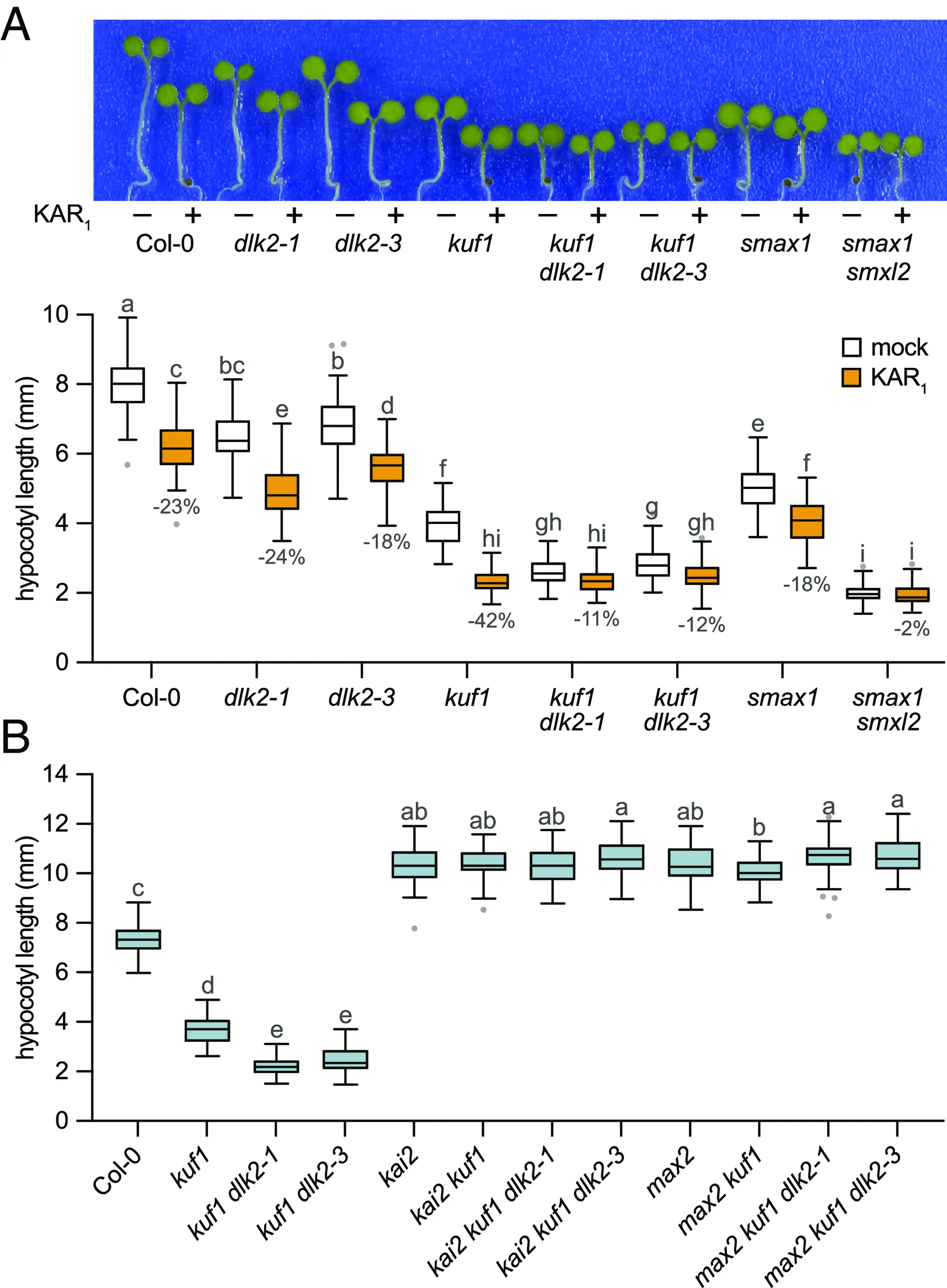
*DLK2* and *KUF1* promote hypocotyl elongation upstream of *KAI2* and *MAX2.* (*A*) Representative image and hypocotyl length of 5-d-old *A. thaliana* seedlings grown in red light on 0.5xMS media with or without 1 µM KAR_1_. Percentages indicate degree of hypocotyl growth inhibition by KAR_1_. *P* < 0.01, two-way ANOVA with post hoc Šídák’s multiple comparisons test; n = 60. (*B*) Epistasis analysis of *KUF1* and *DLK2* with KAR/KL signaling genes *KAI2* and *MAX2*. Hypocotyl length of 5-d-old seedlings grown in red light on 0.5xMS media. *P* < 0.01, one-way ANOVA with post hoc Dunnett’s T3 multiple comparisons test; n = 39 to 60. Box plots shown with Tukey’s whiskers. Letters indicate statistical groups.

We hypothesized that *DLK2* participates in negative feedback regulation of KAR/KL signaling but its role in *A. thaliana* has been obscured by redundant activity from *KUF1*. This led us to test whether combining *kuf1* and *dlk2* mutations affects the hyperactive KAR/KL response phenotypes that are already observed in *kuf1* plants. Under red light, we found that the hypocotyls of *dlk2* seedlings showed a minor (~17%) but significant reduction in length compared to WT (Col-0 ecotype), and responded to KAR_1_, KAR_2_, and *rac*-GR24 treatments similarly to WT (Fig. 1*A* and *SI Appendix,* Fig. S1). In contrast, *kuf1* hypocotyls were 50% shorter than WT and showed a stronger response than WT to KAR_1_ as previously reported (Fig. 1*A*) (43). Strikingly, *kuf1 dlk2* hypocotyls were significantly shorter than *kuf1* and *smax1*, and nearly as short as *smax1 smxl2*, which may be considered to have maximal KAR/KL responses. This suggested that *dlk2* synthetically enhances *kuf1* during seedling photomorphogenesis.

We used an epistasis test to determine whether the short hypocotyl phenotype of *kuf1 dlk2* is dependent on KAR/KL signaling. Previously, we observed that the hypocotyls of *kuf1 kai2* and *kuf1 max2* seedlings have the same length as the KAR/KL-insensitive single mutants *kai2* and *max2* (43). Similarly, the hypocotyls of *kuf1 dlk2 kai2* and *kuf1 dlk2 max2* seedlings had the same length as *kai2* and *max2* (Fig. 1*B*). This masking of the *kuf1 dlk2* phenotype by *kai2* and *max2* suggests that *KAI2* and *MAX2* act genetically downstream of *KUF1* and *DLK2*. Like *KUF1*, *DLK2* expression is positively regulated by KAR/KL signaling in *Arabidopsis* (7). Therefore, we hypothesize that *DLK2* acts in a negative feedback loop that attenuates KAR/KL signaling. Because *dlk2* enhances *kuf1*, the DLK2-mediated feedback loop may operate through a different pathway than *KUF1*.

**Fig. 2.**
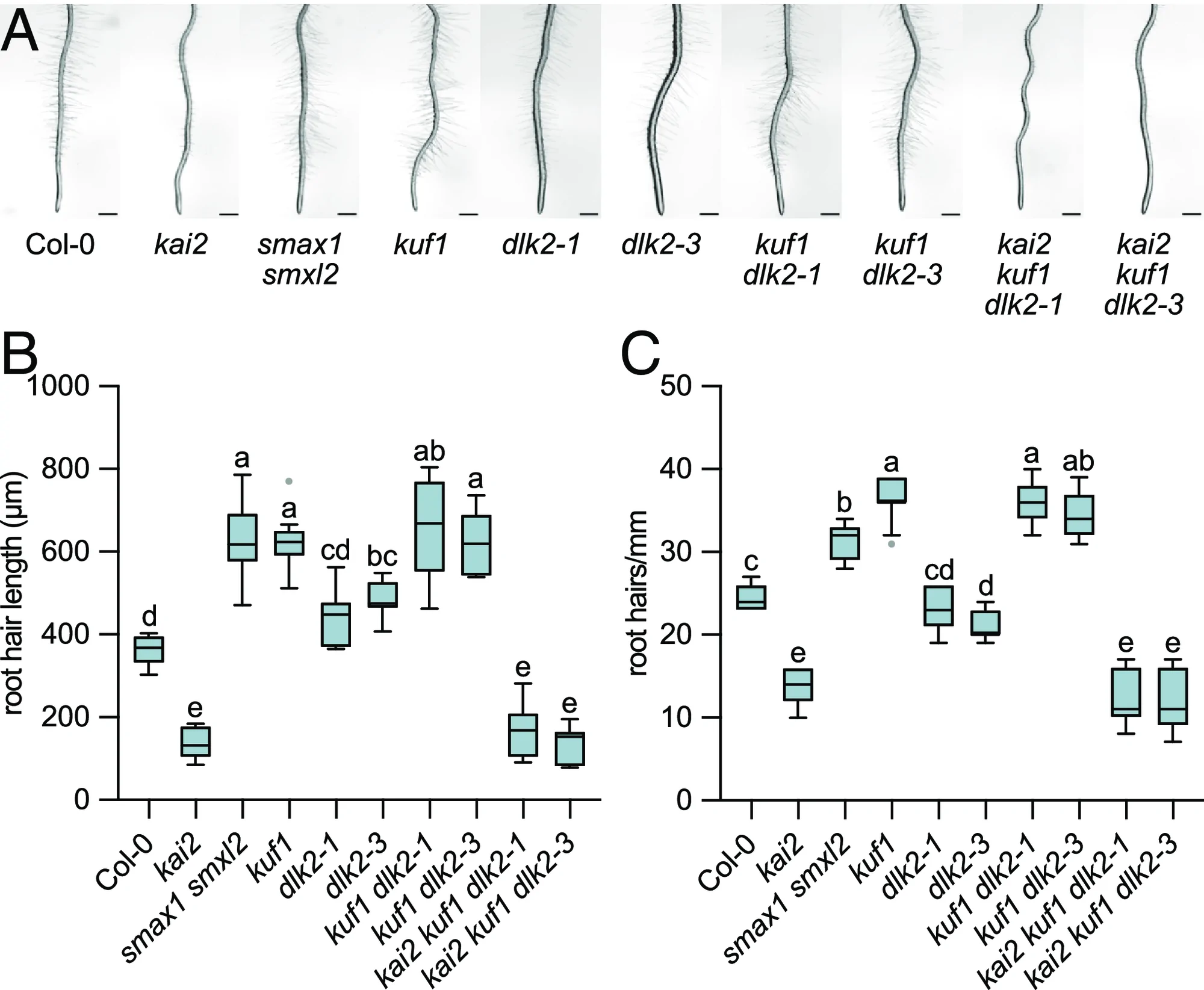
*DLK2* inhibits root hair length but not density. (*A*) Representative images of root tips of 5-d-old *A. thaliana* seedlings. (Scale bar, 500 µm.) (*B*) Root hair length and (*C*) root hair density of 5-d-old seedlings. For each seedling, the mean length or density of all root hairs visible between 2 to 3 mm above the root tip was determined. *P* < 0.01, one-way ANOVA with post hoc Dunnett’s T3 multiple comparisons test; n = 11 seedlings. Box plots shown with Tukey’s whiskers. Letters indicate statistical groups.

To determine whether *DLK2* influences KAR/KL signaling in seedling developmental processes other than hypocotyl growth, we examined its role in root hair development. As observed previously, root hair length and root hair density were reduced in *kai2* and increased in *kuf1* and *smax1 smxl2* seedlings (Fig. 2) (6, 43). Root hair length was increased in *dlk2* seedlings relative to WT, although not as much as in *kuf1* or *smax1 smxl2* (Fig. 2*B* and *SI Appendix,* Fig. S2 *A* and *B*). Root hair density was not affected in *dlk2* seedlings, however (Fig. 2*C* and *SI Appendix,* Fig. S2*C*). In contrast to its effects on hypocotyl elongation, *dlk2* did not enhance the root hair length or root hair density phenotypes of *kuf1* (Fig. 2). This may be because the developmental response of root hairs to hyperactive KAR/KL signaling has already reached its maximum in *kuf1*, as suggested by the similar root hair phenotypes of *kuf1* and *smax1 smxl2*. As observed for hypocotyl elongation, *kai2* was epistatic to *kuf1 dlk2* for both root hair traits (Fig. 2).

We also tested whether the developmental responses of root hairs to KAR_2_ were regulated by *DLK2* (*SI Appendix,* Fig. S2). KAR_2_ caused the root hairs of *dlk2* seedlings to grow to the same, putatively maximized length as *kuf1*, although the degree of growth increase for *dlk2* root hairs (~36%) was not as much as for WT (~52%) (*SI Appendix,* Fig. S2*B*). In contrast, KAR_2_ caused a significantly stronger increase in root hair density in *dlk2* seedlings (~54%) than in WT (30%) (*SI Appendix,* Fig. S2*C*). Altogether, these observations show that *DLK2* inhibits KAR/KL responses in multiple aspects of seedling development, although to a lesser extent than *KUF1*.

**Fig. 3.**
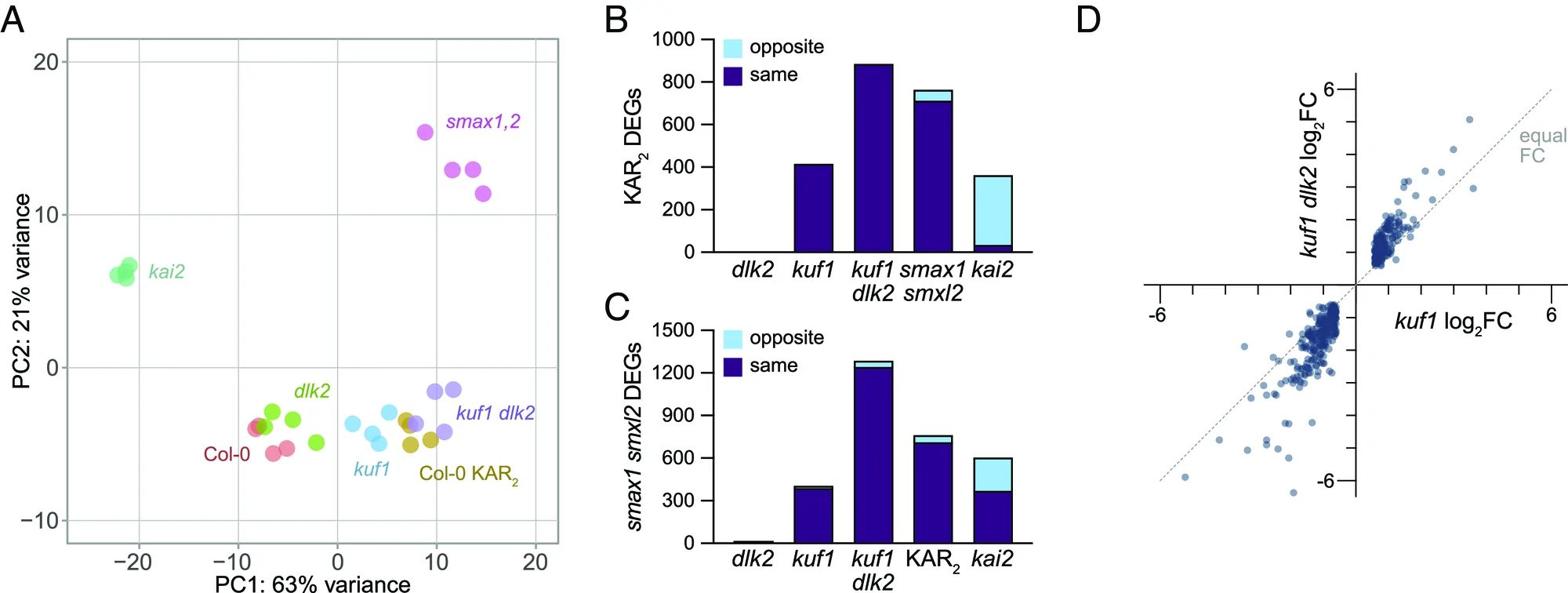
*kuf1* and *dlk2 s*ynthetically enhance KAR/KL-regulated expression. RNA-seq analysis of 5-d-old seedlings grown in red light on 0.5xMS media. n = 4 pools of seedlings. (*A*) Principal component analysis of normalized, mapped-read counts. (*B*) Number of DEGs in *dlk2-3*, *kuf1-1*, *kuf1-1 dlk2-3*, *smax1 smxl2*, and *kai2* (*htl-3*) seedlings that overlap with 1,139 DEGs from KAR_2_-treated WT seedlings. Number of genes with opposite directionality of differential expression (i.e., upregulated in one DEG set and downregulated in the other) or with the same directionality (i.e., upregulated or downregulated in both DEG sets) are indicated within each bar. (*C*) As for (*B*), but with comparisons of DEG sets to 3,579 *smax1 smxl2* DEGs. (*D*) Scatter plot of the log_2_-normalized fold-changes (FC) for 440 DEGs found in both *kuf1* and *kuf1 dlk2*. The dashed line indicates the position of equal FC.

To complement our analysis of *DLK2* function in the development of *Arabidopsis* seedlings, we investigated the effects of *dlk2* on KAR/KL-responsive gene expression (Fig. 3). We performed RNA-seq analysis of WT, *kuf1-1*, *dlk2-3*, and *kuf1-1 dlk2-3* seedlings grown under red light (*SI Appendix,* Table S1). At the same time, we analyzed the transcriptomes of *kai2*, *smax1 smxl2*, and WT treated with 1 μM KAR_2_, which can be used to identify KAR/KL-regulated genes. Principal component analysis showed a distribution of the transcriptomes along the first principal component axis, which accounts for the greatest variance in the data, that is consistent with a spectrum of increasing KAR/KL signaling, ranging from *kai2* (no KAR/KL signaling) < WT and *dlk2* < *kuf1* < WT treated with KAR_2_ < *kuf1 dlk2* < *smax1 smxl2* (maximal KAR/KL signaling) (Fig. 3*A*). We identified differentially expressed genes (DEGs; criteria: absolute fold-change (FC) ≥ 1.5 and false discovery rate-adjusted *P*-value ≤ 0.05) for each mutant genotype or KAR_2_ treatment compared to mock-treated WT (*SI Appendix,* Table S2 and Dataset S1). We observed only 17 DEGs in *dlk2*, indicating this mutation alone had little or no effect on the seedling transcriptome. In contrast, 528 DEGs were found in *kuf1*, 1846 DEGs in *kuf1 dlk2*, 1453 in *kai2*, 3579 DEGs in *smax1 smxl2*, and 1139 DEGs from KAR_2_ treatment.

To determine whether the *kuf1* and *kuf1 dlk2* transcriptomes indicate enhanced KAR/KL signaling, we looked for overlap with DEGs identified in KAR_2_-treated WT and *smax1 smxl2* seedlings (Fig. 3 *B* and *C*). Among the KAR_2_-responsive DEGs, 418 (36.7%) were also identified as DEGs in *kuf1* and 887 (77.9%) were DEGs in *kuf1 dlk2* (Fig. 3*B*). All *kuf1* and *kuf1 dlk2* DEGs had the same direction of expression change as with KAR_2_ treatment (i.e., DEGs were upregulated in both datasets, or downregulated in both datasets). Therefore, the *kuf1 dlk2* and, to a lesser degree, *kuf1* transcriptomes reflect a state of increased KAR/KL signaling. Most (93.2%) of the 765 *smax1 smxl2* DEGs that overlapped with KAR_2_ DEGs also changed expression in the same direction as KAR_2_ DEGs. In contrast, but as expected for KAR/KL-regulated genes, most (90.1%) of the 364 overlapping *kai2* and KAR_2_ DEGs changed expression in opposite directions (i.e., upregulated in one dataset and downregulated in the other).

We observed a similar pattern when comparing *kuf1* and *kuf1 dlk2* DEGs to *smax1 smxl2* DEGs, providing further evidence that *kuf1 dlk2* has enhanced KAR/KL signaling compared to *kuf1* (Fig. 3*C*). However, we unexpectedly found that many (61.4%) of the 606 overlapping *smax1 smxl2* and *kai2* DEGs changed expression in the same direction. This observation was surprising because *smax1 smxl2* and *kai2* putatively represent opposite states of KAR/KL signaling activity: constitutively on and constitutively off, respectively. The similarity in a subset of the *smax1 smxl2* and *kai2* transcriptomes might be due to hormesis, a biphasic dose–response in which a low dose (in this case, of KAR/KL signaling) produces a stronger or opposite effect than a higher dose. For example, high concentrations of auxin restrict hypocotyl cell elongation, while lower concentrations of auxin promote elongation (58). Consistent with hormesis, more than half of the DEGs that were similarly regulated in *smax1 smxl2* and *kai2* showed opposite regulation in KAR_2_-treated seedlings and/or the less extreme KAR/KL signaling mutants *kuf1* and *kuf1 dlk2* (*SI Appendix,* Fig. S3). Additional examples of genes that have similar differential regulation in *kai2a kai2b* and *smax1* mutants can be found in *Lotus japonicus* roots (59).

As another test of whether constitutive KAR/KL responses are stronger in *kuf1 dlk2* seedlings than *kuf1*, we compared the magnitudes of differential expression for the 440 DEGs that are common to both datasets. The vast majority of these DEGs showed a larger FC in expression in *kuf1 dlk2* than in *kuf1* (Fig. 3*D*). These observations collectively demonstrate that *dlk2* enhances *kuf1* effects on KAR/KL signaling, despite having little effect alone.

### DLK2 Promotes SMAX1 Accumulation.

A direct consequence of KAI2-SCF^MAX2^ activation is polyubiquitination and degradation of SMAX1 and, in *Arabidopsis*, its paralog SMXL2 (9, 23, 31). This led us to investigate whether *DLK2* contributes to feedback regulation of KAR/KL signaling by influencing SMAX1 protein abundance.

**Fig. 4.**
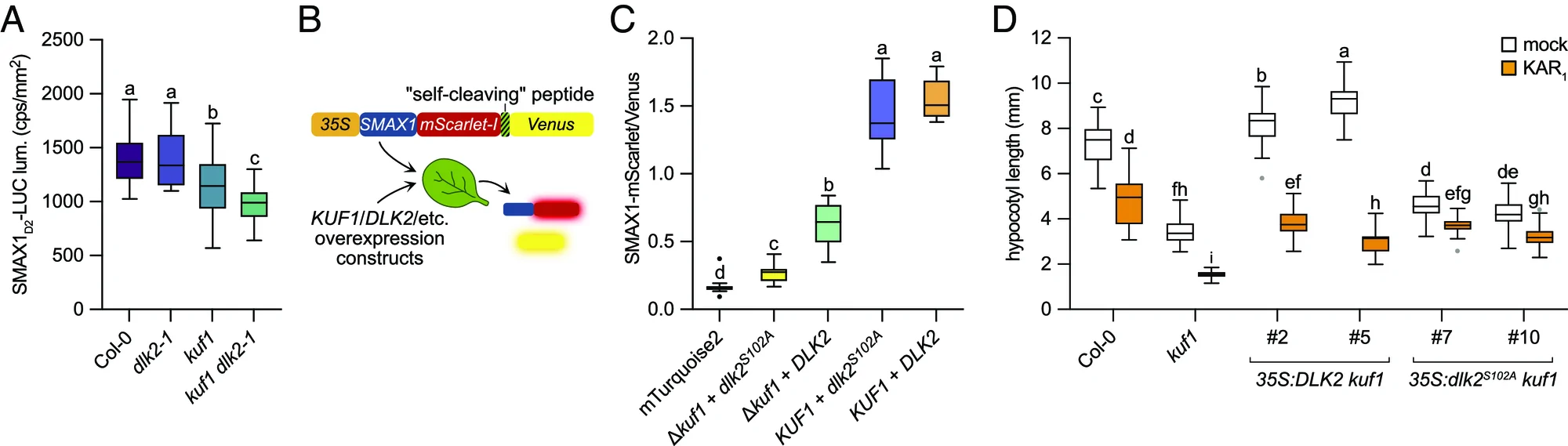
DLK2 promotes SMAX1 protein abundance redundantly with KUF1. (*A*) Relative luminescence (arbitrary units normalized by cotyledon and leaf surface area for each seedling) from a SMAX1_D2_-LUC translational reporter expressed in 9-d-old seedlings grown in 16 h white light:8 h dark photoperiod. *P* < 0.01, one-way ANOVA with post hoc Dunnett’s T3 multiple comparisons test; n = 36. (*B*) Diagram of ratiometric translational reporter system used in C) for monitoring the effects of coexpressed genes on SMAX1 abundance after transient expression in *Nicotiana benthamiana* leaves. (*C*) Relative fluorescence of SMAX1-mScarlet to Venus in *N. benthamiana* leaves coinfiltrated with constructs that overexpress *mTurquoise2*, *KUF1*, *DLK2*, *∆kuf1*, or *dlk2^S102A^*. *∆kuf1* produces the N-terminal 46 aa of KUF1 only due to a deletion. *dlk2^S102A^* has a mutation in a catalytic triad residue. *P* < 0.01, one-way ANOVA with post hoc Dunnett’s T3 multiple comparisons test; n = 14 leaf discs. (*D*) Hypocotyl length of 5-d-old seedlings grown in red light on 0.5xMS medium containing 1 μM KAR_1_ or solvent control. Measurements of two independent homozygous transgenic lines for *35S:DLK2* or *35S:dlk2^S102A^* in the *kuf1* background are shown. Data in (*D*) are duplicated in *SI Appendix,* Fig. S6 for comparison of transgene effects in other genetic backgrounds. *P* < 0.01, two-way ANOVA with post hoc Šídák’s multiple comparisons test; n = 32 to 45. Box plots shown with Tukey’s whiskers. Letters indicate statistical groups.

First, we examined the effect of *dlk2* on a translational reporter that fuses the C-terminal D2 domain of SMAX1 to firefly luciferase (23). The stably integrated transgene was crossed into different genetic backgrounds in *Arabidopsis* to control for positional effects of T-DNA integration. Luminescence from the SMAX1_D2_-LUC reporter was similar in WT and *dlk2* seedlings. However, luminescence was reduced in *kuf1* seedlings and further reduced in *kuf1 dlk2* seedlings (Fig. 4*A*). This implies that *DLK2* promotes SMAX1 protein abundance redundantly with *KUF1*, but to a lesser degree.

Luminescence from the SMAX1_D2_-LUC reporter declined faster in *kuf1 dlk2* seedlings than in WT following KAR_1_ treatment (*SI Appendix,* Fig. S4). In contrast, SMAX1_D2_-LUC responses to KAR_2_ and *rac*-GR24 in *kuf1 dlk2* remained similar to WT. The hypersensitive response of *kuf1 dlk2* to KAR_1_ specifically seems likely to be an effect of *kuf1*, consistent with hypocotyl growth assays, that is further enhanced by lowered SMAX1 abundance.

Next, we examined the effects of *KUF1* and *DLK2* overexpression in a transient expression system. We monitored SMAX1 protein abundance with a ratiometric translational reporter that is expressed in *N. benthamiana* leaves (23, 60). In this system, a SMAX1-mScarlet-I translational fusion is cotranscribed with the coding sequences for a modified, “self-cleaving” 2A peptide from foot-and-mouth disease virus and a yellow fluorescent protein, Venus (Fig. 4*B*). During translation, ribosomal “skipping” of a peptide bond near the C-terminal end of 2A causes separation of the SMAX1-mScarlet-I and Venus proteins. By measuring fluorescence from both proteins, Venus then serves as a reference for monitoring relative changes in SMAX1-mScarlet-I abundance. As negative controls, we tested expression of a *kuf1* mutant coding sequence that only produces the first 46 aa of the 395 aa protein (Δ*kuf1*), and a *dlk2^S102A^* mutant in which a catalytic triad residue is disrupted. Overexpression of either *KUF1* or *DLK2* caused an increase in the ratio of mScarlet-I to Venus fluorescence that indicates enhanced accumulation of SMAX1-mScarlet-I protein (Fig. 4*C*). However, *KUF1* overexpression had a stronger effect than *DLK2* overexpression on the relative abundance of the SMAX1 reporter.

We also tested the effect of *KUF1* and *DLK2* overexpression on the abundance of a SMXL7 ratiometric reporter, which is subject to degradation in response to SL but not KAR/KL signaling. In contrast with SMAX1, we found no evidence that KUF1 or DLK2 affect SMXL7 reporter stability in *N. benthamiana* (*SI Appendix,* Fig. S5). This suggests that KUF1 and DLK2 regulate KAR/KL signaling specifically rather than SCF^MAX2^-dependent signaling generally.

We reasoned that if DLK2 and KUF1 both reduce SMAX1 degradation (or promote its abundance), that DLK2 may be able to substitute for a loss of KUF1 activity even if it operates through a different mechanism. We found that overexpression of *DLK2* rescued the hypocotyl length of *kuf1* seedlings (Fig. 4*D*). In comparison, overexpression of *dlk2^S102A^* at even higher levels caused only a weak increase in hypocotyl elongation (Fig. 4*D* and *SI Appendix*, Fig. S6). These observations show that DLK2 function does not require KUF1 but is mostly dependent on an intact catalytic triad. Notably, *DLK2* overexpression did not rescue the hypersensitive response of *kuf1* seedlings to KAR_1_ (*SI Appendix,* Fig. S6). This suggests that while DLK2 participates in feedback regulation of KAR/KL signaling, it does so in a different manner than KUF1, perhaps by affecting KL abundance specifically and not KAR_1_ metabolites.

We also tested the effects of *DLK2* overexpression in WT, *dlk2*, and *kuf1 dlk2* backgrounds (*SI Appendix,* Fig. S6). We consistently observed that *DLK2* overexpression caused a significant increase in hypocotyl elongation, while *dlk2^S102A^* overexpression at similar levels had weaker or nonstatistically significant effects on hypocotyls. *DLK2* overexpression had the least effect on hypocotyl growth in the *dlk2* mutant, which might be due to lower expression of the *DLK2* transgene than in the other genetic backgrounds or because expression of native *DLK2* is somehow important.

### The Molecular Function of DLK2.

**Fig. 5.**
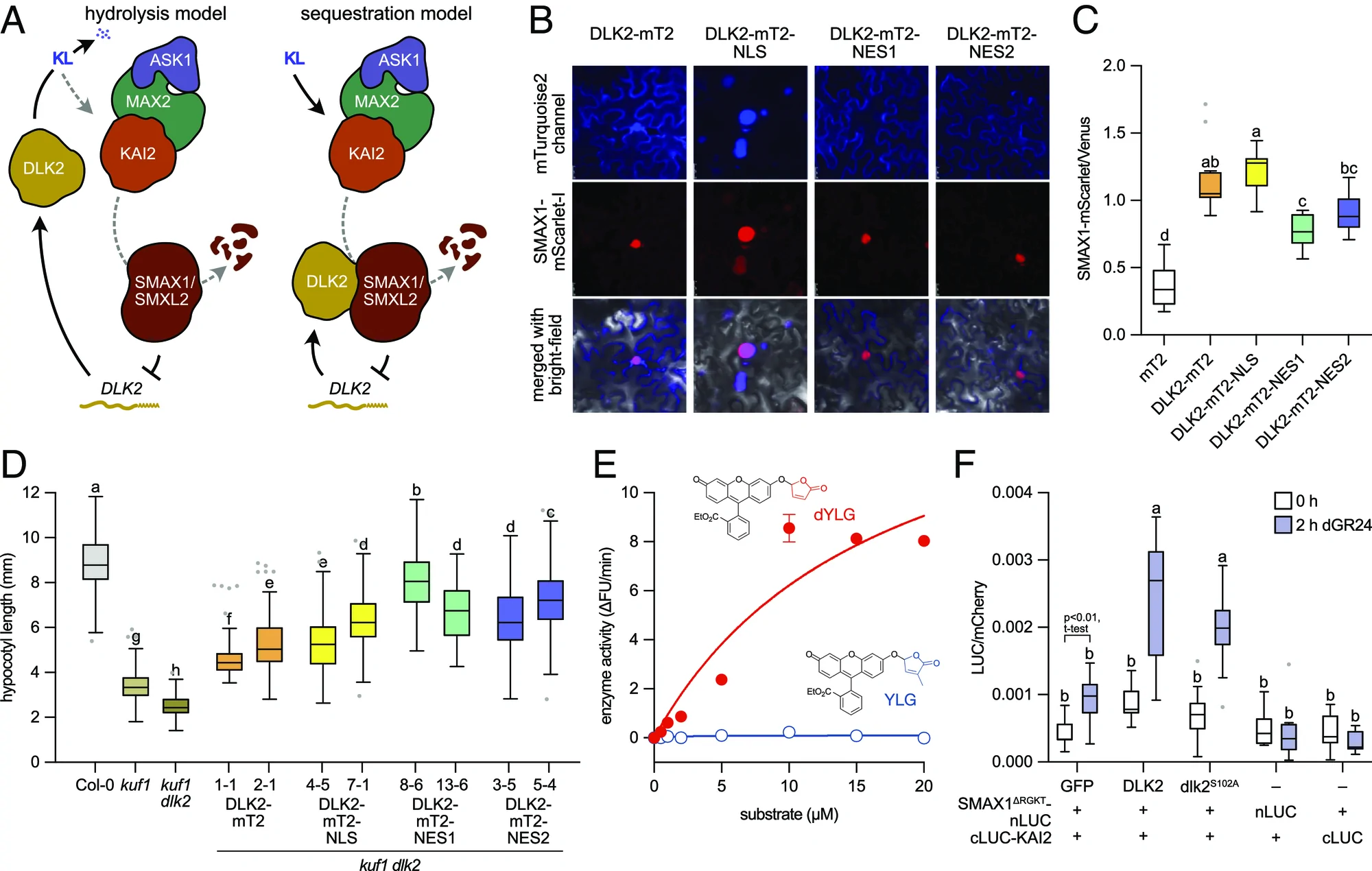
DLK2 likely acts through hydrolysis of KAI2 ligands. (*A*) Two hypotheses for feedback regulation of KAR/KL signaling by DLK2. In the hydrolysis model, DLK2 catabolizes one or more signals that activate KAI2. In the sequestration model, DLK2 association with a core KAR/KL signaling component (e.g., SMAX1/SMXL2) inhibits SMAX1/SMXL2 polyubiquitination by KAI2-SCF^MAX2^ and degradation. DLK2 expression is inhibited by SMAX1/SMXL2. (*B*) Confocal fluorescence microscopy of transiently coexpressed translational reporters of DLK2 and SMAX1 in *N. benthamiana* mesophyll. mT2, mTurquoise2; NLS, nuclear localization sequence; NES, nuclear exclusion sequence. (*C*) Relative fluorescence of SMAX1-mScarlet to Venus in *N. benthamiana* leaves coinfiltrated with constructs that overexpress *mTurquoise2*, *DLK2-mT2*, or *DLK2-mT2* with altered subcellular localization. *P* < 0.01, one-way ANOVA with post hoc Dunnett’s T3 multiple comparisons test; n = 12 leaf discs. (*D*) Hypocotyl length of 5-d-old seedlings grown in red light on 0.5xMS medium. Measurements of two independent homozygous transgenic lines for *35S:DLK2* fusions in the *kuf1-1 dlk2-1* background are shown. *P* < 0.01, one-way ANOVA with post hoc Dunnett’s T3 multiple comparisons test; n = 119 to 235 seedlings. (*E*) In vitro hydrolysis of profluorescent reporter molecules Yoshimulactone Green (YLG) and desmethyl Yoshimulactone Green (dYLG) by 4 μg SUMO-AtDLK2 in 100 mM HEPES, 150 mM NaCl, pH 7. Blank-subtracted mean ± SEM of n = 3 technical replicates. (*F*) Split-luciferase assays for *DLK2* effect on KAI2-SMAX1^DRGKT^ protein–protein interactions in *N. benthamiana* leaves. Luminescence was normalized to fluorescence from an mCherry reporter used as a transformation control. Leaf discs were measured before and after 2 h treatment with 10 μM dGR24. *P* < 0.05, two-way ANOVA with post hoc Šídák’s multiple comparisons test; n = 8 to 15 leaf discs. Box plots shown with Tukey’s whiskers. Letters indicate statistical groups.

We investigated how DLK2 may attenuate KAR/KL signaling. As discussed above, DLK2 is closely related to the enzyme-receptors D14 and KAI2, which have both hydrolytic and ligand-dependent signal transduction activities. DLK2 has conserved catalytic triad residues that are conserved and important for its function (Fig. 4*D* and *SI Appendix,* Fig. S6), like D14 and KAI2, suggesting that it retains enzymatic activity. However, DLK2 lacks conservation of several surface residues that mediate D14 and KAI2 interactions with MAX2 (47, 50). This suggests that DLK2 does not engage in SCF^MAX2^-dependent signaling. These observations led us to propose two hypotheses for DLK2 function that are not necessarily mutually exclusive (Fig. 5*A*). In the “hydrolysis model,” DLK2 may inhibit KAI2-SCF^MAX2^-mediated degradation of SMAX1/SMXL2 by acting as a hydrolase that catabolizes KAI2 ligand(s) but does not engage in signal transduction directly. In the “sequestration model,” DLK2 may compete with KAI2 for interactions with SMAX1, protecting SMAX1 from being targeted by the KAI2-SCF^MAX2^ complex. Alternatively, DLK2 might sequester KAI2, preventing it from participating in a signaling complex. Similar to KAI2 and D14, DLK2 protein–protein interactions may be allosterically regulated, for example occurring only after binding and/or hydrolyzing a ligand.

Lacking knowledge of KL or DLK2 ligand(s), we reasoned that these hypotheses could be tested by determining whether DLK2 function depends upon its subcellular localization. MAX2 is nuclear-localized, as are SMAX1 and its other SMXL family protein targets (23, 39, 40, 61–64). Like KAI2 and D14, however, DLK2 protein is found in the nucleus and the cytoplasm (25, 47, 53, 64). If DLK2 protected SMAX1 from KAI2-SCF^MAX2^-mediated degradation, for example by moving it outside the nucleus, SMAX1 would be prevented from regulating transcription. This would produce a similar effect as SMAX1 degradation. Therefore, the sequestration model requires DLK2 to colocalize with SMAX1 in the nucleus in order to protect SMAX1 from degradation while also preserving its access to DNA and transcriptional regulators. However, if DLK2 acts via KL hydrolysis, it may be able to inhibit KAI2 signaling and SMAX1 degradation whether it is located inside or outside the nucleus.

This led us to test the function of nuclear-localized and nuclear-excluded forms of DLK2. Fusion of Venus with an N7 nuclear localization sequence (NLS) to the C-terminus of D14 results in nucleus-specific localization of D14; moreover, the fusion protein remains functional (64, 65). We created a similar C-terminal fusion of the fluorescent protein mTurquoise2 and N7 NLS to DLK2 (here, DLK2-mT2-NLS) (66). To make a form of DLK2-mTurquoise2 that is excluded from the nucleus, we added a 20-aa, C-terminal motif from AtRanBP1b (here, NES1) that is homologous to a sequence that confers cytoplasm-specific localization of GFP in plant cells (67). Separately, we added a 14-aa motif (here, NES2) that retains GFP in the cytoplasm of mammalian cells when used as either an N-terminal or C-terminal fusion (68). To test the function of these NLS and NES motifs, we performed transient expression of the DLK2 fusion proteins in *N. benthamiana* leaves, coexpressing SMAX1-mScarlet-I as a nuclear marker. DLK2-mT2 showed putatively nucleocytoplasmic localization. In contrast, DLK2-mT2-NLS had nucleus-specific localization that overlapped with SMAX1, while DLK2-mT2-NES1 and DLK2-mT2-NES2 were localized outside the nucleus (Fig. 5*B*).

We then investigated how coexpression of the differently localized DLK2 fusion proteins affected the abundance of the SMAX1 ratiometric reporter in *N. benthamiana* leaves. As observed with DLK2, DLK2-mT2 caused increased accumulation of the SMAX1 reporter. DLK2-mT2-NLS had a similar effect (Fig. 5*C*). Importantly, both forms of nuclear-excluded DLK2-mT2 fusion proteins also caused an increase in SMAX1 reporter abundance, although not quite as effectively as DLK2-mT2-NLS (Fig. 5*C*). This suggests that colocalization with SMAX1 in the nucleus is not required for DLK2 to promote SMAX1 accumulation.

To validate these results, we overexpressed the *DLK2* fusions in the *Arabidopsis kuf1 dlk2* background. Similar patterns of subcellular localization were observed for the DLK2 fusion proteins in *Arabidopsis* roots as in *N. benthamiana* mesophyll (*SI Appendix*, Fig. S7). DLK2-mT2-NES1 and DLK2-mT2-NES2 caused a significant increase in hypocotyl elongation of *kuf1 dlk2* seedlings, as did DLK2-mT2 and DLK2-mT2-NLS (Fig. 5*D*). This confirms that nuclear localization of DLK2 is not necessary for its function, providing some support for the hydrolysis model of DLK2 action.

Because the actual substrate(s) of DLK2 has not been identified, it is not possible to assess its true enzymatic activity. DLK2 has very weak hydrolytic activity on (–)-5-deoxystrigol, an unnatural enantiomer of a SL, but not on (+)-5-deoxystrigol itself (47). Desmethyl butenolide compounds, which lack the methyl group found on the butenolide D-ring of all known SLs, are better agonists of KAI2 than equivalent methyl butenolides, suggesting they more closely resemble KL(s) (13, 24, 69, 70). This led us to test the in vitro hydrolytic activity of SUMO-tagged *Arabidopsis* DLK2 against the profluorescent butenolide molecules Yoshimulactone Green (YLG) and desmethyl YLG (dYLG), which have been used to assay KAI2/HTL activity (24, 71). *Arabidopsis* DLK2 hydrolyzed dYLG, but not YLG (Fig. 5*E*), potentially indicating specificity for a KL-like substrate that would be consistent with the hydrolysis model.

To further examine the sequestration model, we tested whether coexpression of DLK2 interfered with KAI2-SMAX1 interaction in a split-luciferase assay. We used a stabilized, SCF^MAX2^-resistant form of SMAX1 in which an Arg-Gly-Lys-Thr (“RGKT” or P-loop) motif has been deleted (SMAX1^ΔRGKT^) to prevent misleading decreases in luciferase signal that might be caused by SMAX1 degradation. As negative controls, we tested the effects of coexpressing GFP or dlk2^S102A^. Treatment with desmethyl-GR24 (dGR24) for two hours in the presence of coexpressed GFP increased the relative luciferase signal from SMAX1^ΔRGKT^-nLUC and cLUC-KAI2 (Fig. 5*F*; *P* < 0.01, Student’s two-tailed *t* test). This implies enhanced interaction between these two proteins that is consistent with KAI2 activation. Coexpression of DLK2 did not reduce the relative luciferase signal either before or after dGR24 treatment (Fig. 5*F*). Coexpression of dlk2^S102A^ had a similar effect as DLK2 (Fig. 5*F*). Similar results were obtained with KAR_1_ treatments as well as for split-luciferase assays of SMXL2-nLUC and cLUC-KAI2 interactions (*SI Appendix,* Fig. S8). Therefore, we did not find any evidence that DLK2 interferes with KAI2-SMAX1 or KAI2-SMXL2 interactions.

We also used split-luciferase assays to test for potential interactions between DLK2 and SMAX1, SMXL2, and KAI2. There was some evidence for interactions between DLK2 and stabilized SMAX1^ΔRGKT^ and SMXL2 that increased over a 3-h treatment with dGR24 (*SI Appendix,* Fig. S9). However, the same interactions occurred with dlk2^S102A^, which has a highly reduced function compared to DLK2 (Fig. 4*D* and *SI Appendix,* Fig. S6). Therefore, interactions with SMAX1/SMXL2, if real, are unlikely to explain how DLK2 works. In contrast, DLK2 showed clear evidence of a stronger interaction than dlk2^S102A^ with KAI2, both in the absence and presence of the KAI2-activating compounds KAR_1_ and dGR24 (*SI Appendix,* Fig. S10). Sequestration of KAI2 could provide a plausible explanation for SMAX1 protection by DLK2 that would also be compatible with DLK2 retaining functionality when excluded from the nucleus. However, KAI2 sequestration by DLK2 would presumably inhibit KAI2-SMAX1 interactions, which we did not observe (Fig. 5*F*). Therefore, we currently favor the hydrolysis model to explain DLK2 function.

### Conservation of DLK2 Function in Angiosperms.

**Fig. 6.**
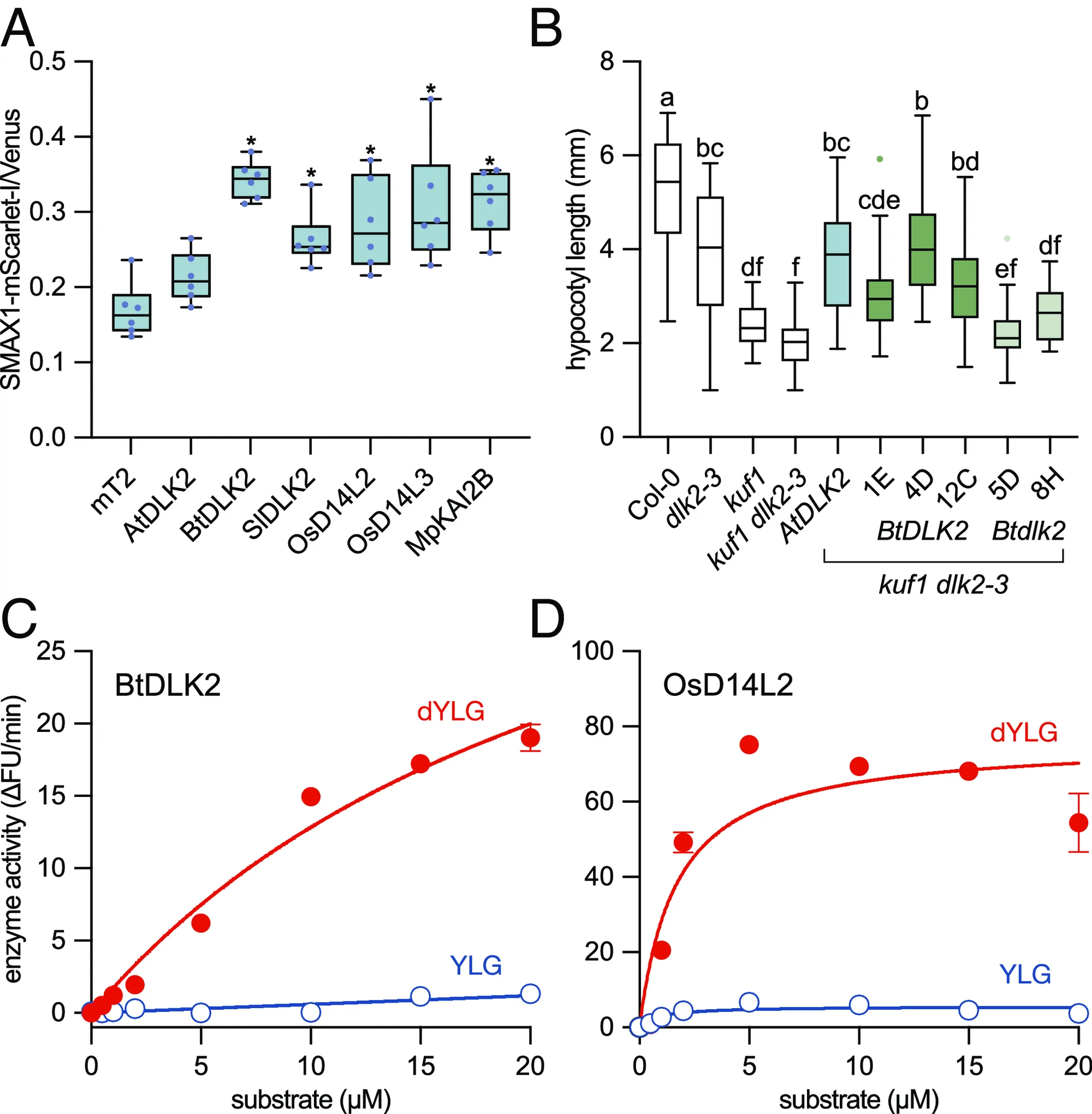
*DLK2* proteins from multiple species have similar activities. (*A*) Relative fluorescence of SMAX1-mScarlet to Venus in *N. benthamiana* leaves coinfiltrated with constructs that overexpress *mTurquoise2* (*mT2*), *A. thaliana DLK2* (*AtDLK2*), *Brassica tournefortii DLK2* (*BtDLK2*), *Solanum lycopersicum DLK2* (*SlDLK2*), *Oryza sativa D14L2* (*OsD14L2*) and *D14L3* (*OsD14L3*), and *Marchantia polymorpha KAI2B* (*MpKAI2B*). *P < 0.01, one-way ANOVA with post hoc Dunnett’s multiple comparisons test of DLK2-like genes versus mT2; n = 6 leaves, average of 5 to 6 leaf discs per leaf. (*B*) Hypocotyl length of 5-d-old *A. thaliana* seedlings grown in red light on 0.5xMS media. Measurements for homozygous transgenic lines of *35S:AtDLK2*, *35S:BtDLK2*, and *35S:Btdlk2^S102A^* in the *kuf1 dlk2-3* background are shown. *P* < 0.01, one-way ANOVA with post hoc Tukey’s multiple comparisons test; n = 21 to 32. Box plots shown with Tukey’s whiskers. Letters indicate statistical groups. (*C*) and D) In vitro hydrolysis of profluorescent reporter molecules YLG and dYLG by 4 μg of (*C*) SUMO-BtDLK2 or (*D*) SUMO-OsD14L2 in 100 mM HEPES, 150 mM NaCl, pH 7. Blank-subtracted mean ± SEM of n = 3 technical replicates.

We investigated whether DLK2 proteins from other plants have similar effects on SMAX1. We used the transient expression system in *N. benthamiana* to analyze *DLK2* genes from *Brassica tournefortii* (*BtDLK2*), tomato (*SlDLK2*), and rice (*OsD14L2* and *OsD14L3*). Like *AtDLK2*, *BtDLK2* expression is KAR-responsive (72)*. OsD14L2* and *OsD14L3* are *DLK2* orthologs that are positively regulated by the KAR/KL signaling pathway (8–10). *OsD14L2* and *OsD14L3* are also highly upregulated in response to arbuscular mycorrhizal colonization, as is *SlDLK2* (48, 56). All four DLK2 proteins caused an increase in the abundance of the SMAX1 ratiometric reporter when coexpressed in *N. benthamiana* leaves (Fig. 6*A*). This suggests that they have conserved functions in the regulation of SMAX1 abundance or stability.

Indeed, similar to *Arabidopsis DLK2*, *BtDLK2* overexpression increased the hypocotyl elongation of *kuf1 dlk2* in a manner that was largely dependent on the serine residue of the catalytic triad (Fig. 6*B*). We also tested the catalytic activities of purified SUMO-tagged BtDLK2 and OsD14L2 proteins on YLG and dYLG. BtDLK2 showed higher enzymatic activity with dYLG substrate than *Arabidopsis* DLK2 and had very low activity on YLG (Fig. 6*C*). The higher activity of BtDLK2 on dYLG might be due to its lacking Phe167, which partially obstructs substrate access to the active site of AtDLK2 (49). Regardless, the hydrolytic activity of BtDLK2 was fully dependent on the catalytic serine residue (*SI Appendix,* Fig. S11*A*). OsDLK2 was even more active on dYLG substrate than BtDLK2 and had low activity on YLG (Fig. 6*D*). Therefore, a preference for hydrolysis of desmethyl butenolide substrates may be a common attribute of DLK2 proteins in angiosperms.

### Possible Evolution of KAR/KL Feedback Regulation from KAI2.

We hypothesized that a DLK2-like function in negative feedback regulation of KAR/KL signaling, whether hydrolysis- or sequestration-based, could evolve in a KAI2 paralog through two steps. First, the KAI2 paralog could acquire a mutation(s) that disrupts its interaction with a signaling partner, i.e. MAX2 or SMAX1/SMXL2, but does not affect its ability to bind and hydrolyze KL. Second, expression of this subfunctionalized *KAI2* paralog could become positively regulated by KAR/KL signaling. Potential examples of this may be found in bryophytes, which lack *DLK2* genes but have evolutionarily diverged *KAI2* homologs in addition to “true”*KAI2* (28, 29). For instance, the liverwort *Marchantia polymorpha* has two *KAI2* paralogs. MpKAI2A works with MpMAX2 to negatively regulate MpSMXL, similar to what is observed in angiosperms (28). *MpKAI2B*, in contrast, has no obvious function based on genetic analysis. But, *MpKAI2B* shows several similarities to *DLK2*. *MpKAI2B* is downregulated in *Mpkai2a* and *Mpmax2* mutants or when a degradation-resistant MpSMXL protein is expressed. Furthermore, *MpKAI2B* expression is upregulated in the *Mpsmxl* mutant (28). Finally, MpKAI2B hydrolyzes GR24*^ent^*^-5DS^ and shows thermal destabilization in the presence of GR24*^ent^*^-5DS^, but not SL-like stereoisomers of GR24, suggesting it might act on a KL substrate (22, 28).

This reasoning led us to test whether MpKAI2B affects SMAX1 abundance in the *N. benthamiana* transient expression assay. We found that *MpKAI2B* coexpression caused a significant increase in the abundance of the SMAX1 ratiometric reporter, similar to DLK2 proteins from *Arabidopsis* and other angiosperms (Fig. 6*A*).

## Discussion

### DLK2 Probably Acts As an Enzyme and Not a Receptor.

DLK2 has been hypothesized to act as a receptor based on its homology to KAI2 and D14. Although signaling via SCF^MAX2^ has been considered unlikely due to poor conservation of MAX2-interacting residues, it has been proposed that DLK2 may modulate gene expression through other protein–protein interactions (47, 48, 50, 56). For example, SlDLK2 in tomato can physically interact with the DELLA protein GIBBERELLIN INSENSITIVE (GAI) (56). DELLA proteins are nuclear-localized members of the GRAS family of transcriptional regulators in plants. Although DELLAs do not bind DNA directly, through protein–protein interactions with a diverse array of transcription factors and transcriptional regulators DELLAs can either activate or repress gene expression (73). Based on the effects of *SlDLK2* misexpression and the important role of DELLAs in root colonization by AMF, the idea arose that SlDLK2 interaction with DELLA may block it from activating the expression of *REDUCED ARBUSCULAR MYCORRHIZA1* (*RAM1*), a master regulator of arbuscule development (56).

We have found, however, that DLK2 function does not require nuclear localization, at least in *Arabidopsis* (Fig. 5*D*). This implies that physical interaction with nuclear-localized factors such as DELLA proteins, MAX2, or SMAX1 is not necessary for DLK2 to enhance SMAX1 stability or regulate downstream responses to KAR/KL. At the same time, nuclear-specific forms of DLK2 are functional, demonstrating that cytoplasmic localization of DLK2 is also not required (Fig. 5 *C* and *D*). This suggests that DLK2 does not inhibit SMAX1 degradation by moving KAR/KL signaling components outside of the nucleus.

Instead of DLK2 acting as a receptor with allosterically regulated protein–protein interactions, we hypothesize that it attenuates KAR/KL signaling through hydrolytic activity (*SI Appendix,* Fig. S12). Supporting this idea, DLK2 function is highly dependent on a catalytic serine residue, and DLK2 acts on desmethyl butenolide substrates that are putatively analogous to KL (Figs. 5*E* and 6 *C* and *D*). In our in vitro assays, the tested dYLG concentrations failed to saturate either SUMO-AtDLK2 or SUMO-BtDLK2, leading to wide, but relatively high estimates of V_max_ and K_m_. This contrasted with SUMO-AtKAI2, which showed substrate saturation (*SI Appendix,* Fig. S11*B*). The estimated V_max_ of BtDLK2 is over three-fold greater than that of AtKAI2, and the estimated V_max_ of AtDLK2 is comparable to AtKAI2 (*SI Appendix,* Table S3). Notably, AtDLK2 and BtDLK2 displayed at least 17-fold greater K_m_ values than AtKAI2, implying lower affinities for dYLG (*SI Appendix,* Table S3). Therefore, DLK2 proteins show the ability to hydrolyze similar substrates as KAI2 at comparable or potentially faster rates, but lack the tight association with these substrates that may be important for signal transduction. The near-linear Michaelis–Menten curves for AtDLK2 and BtDLK2 indicate that both enzymes can catalyze rapid hydrolysis of dYLG, but only when the substrate concentration is high. In a biological context, this is typical of enzymes with roles in regulatory metabolism that may not be highly active unless there is an excess of substrate. A catabolic enzyme that shows reduced activity when KL is scarce and heightened activity when KL is in abundance would be well-suited to maintain KL homeostasis.

### Catabolic Mechanisms for Feedback Regulation of KL and SL.

Another mechanism for feedback regulation of KAR/KL signaling has been described in the bryophyte *Marchantia polymorpha* (74). Like *MpKAI2B*, *DIENELACTONE HYDROLASE LIKE PROTEIN1* (*DLP1*) expression is upregulated in the *Mpsmxl* mutant, downregulated in *Mpkai2a* and *Mpmax2*, and induced by (–)-GR24 treatment in a manner that is dependent on KAR/KL signaling. Loss of *DLP1* induces *MpKAI2B* expression, suggesting more active KAR/KL signaling. In contrast, *DLP1* overexpression represses *MpKAI2B* transcript abundance and causes upward growth of thalli that is similar to the *Mpkai2a* and *Mpmax2* mutants. The effects of *DLP1* misexpression are mostly dependent on the MpKAI2A-SCF^MpMAX2^-MpSMXL pathway, suggesting that DLP1 acts in an inhibitory manner upstream of KL perception. DLP1 has enzymatic activity on *trans*-dienelactone, which is a butenolide compound like KARs and SLs. Therefore, it may be that DLP1 is involved in KL breakdown, similar to DLK2. However, *DLP1* homologs in *Arabidopsis* do not appear to have similar roles in attenuation of KAR/KL signaling (74). Thus, DLP1 and DLK2 may have analogous functions in KL homeostasis but varying physiological significance for different plant lineages. In *Marchantia polymorpha*, DLP1 activity may explain the lack of a detectable *Mpkai2b* mutant phenotype. In *Arabidopsis*, perhaps other sources of redundancy in KL catabolism in addition to KUF1 activity could explain why *dlk2* mutant phenotypes are weak.

Negative feedback control of SL signaling also occurs through SL catabolism. It has been proposed that the hydrolytic activity of D14 is important for SL homeostasis rather than the initiation of SL signal transduction (38). Recently, cryo-EM was used to characterize the structure of ShHTL7 (a KAI2 paralog and SL receptor from the root parasitic plant *Striga hermonthica*) in complex with ShMAX2, *Arabidopsis* Skp1, and *Arabidopsis* SMAX1. Following SL-cleavage and association with MAX2, the active, covalently modified form of ShHTL7 is stabilized through the exclusion of water from the hydrophobic active site and a conformational change that disrupts the catalytic triad (51). This finding fits with prior observations that D14 acts like a “one-shot” enzyme that has an initially fast hydrolysis reaction followed by a long reset period (37). Overall, this makes SL hydrolysis by D14 slow and inefficient (35, 52). A more likely candidate for carrying out physiologically meaningful SL catabolism is a carboxylesterase protein, CXE15. In *Arabidopsis*, AtCXE15 efficiently hydrolyzes both canonical and noncanonical SLs, showing much stronger activity than AtD14 (75, 76). *AtCXE15* expression is induced by synthetic SL application, consistent with a mechanism for feedback regulation (75). CXE15 orthologs from several other angiosperms also catabolize SL (75). The existence of these mechanisms for KL and SL catabolism suggests that the ability of KAI2 and D14 to hydrolyze their own ligands is inadequate for homeostatic control. We argue that ligand hydrolysis by KAI2 and D14 is an integral part of the signal transduction mechanism, rather than an effective way to limit KL and SL levels.

This study raises the question of why multiple mechanisms for feedback regulation of KAR/KL signaling have evolved in plants. Although KUF1 and DLK2 act in different ways—putatively by inhibiting KAR/KL metabolism and catabolizing KAI2 agonists respectively—the consequences for SMAX1 stability are similar. The selective advantage of having redundant mechanisms is unclear at first glance. We hypothesize that there may be differences in the timing (i.e., fast versus slow response), intensity/efficacy, location (e.g., tissue-specificity), and/or concentration dependence of these two feedback mechanisms. Although *DLK2* appears to have a minor role in comparison to *KUF1* during *Arabidopsis* seedling development, it may have greater or unique physiological significance for other developmental stages, growth processes, or species. For example, *DLK2* expression is induced by AMF in tomato, *Brachypodium distachyon*, and rice (13, 48, 56). In addition, *DLK2* misexpression affects AMF colonization of tomato roots (56). Thus, it will be particularly interesting to explore the respective roles of *DLK2* and *KUF1* in the establishment, persistence, and cellular distribution of arbuscular mycorrhization.

### Evolution of Diverse Functions Among the KAI2 Family of α/β-Hydrolases.

KAI2B in *Marchantia polymorpha* shows similarity to DLK2 in angiosperms both in terms of its transcriptional regulation by the KAR/KL pathway and its ability to stabilize a transiently expressed AtSMAX1 reporter. *MpKAI2B* and *DLK2* may have evolved in similar ways through subfunctionalization of a *KAI2* paralog. DLK2 proteins form part of a superclade referred to as “DDK” (D14/DLK2/KAI2) that includes the D14 clade and clades of uncharacterized D14-LIKE proteins (50). The DDK and eu-KAI2 (true KAI2) superclades diverged following a deep duplication event near the base of the land plant lineage, with eu-KAI2 having a broader distribution in extant species (50). The DDK superclade illustrates the malleability of KAI2 evolution. While D14 functions similarly to KAI2 through interactions with SCF^MAX2^ and SMXL proteins, its ligand recognition was neofunctionalized from KL to SL (50, 77). A similar neofunctionalization event occurred more recently within the parasitic Orobanchaceae, producing receptors for SLs and other host-derived germination stimulants from divergent KAI2 paralogs (KAI2d) (78–80). DLK2 appears to have undergone a different route of specialization, putatively through subfunctionalization that lost MAX2-dependent signal transduction capacity while retaining ligand-specificity. By imposing feedback regulation of KAR/KL signaling, DLK2 may be considered a functionally antagonistic paralog of KAI2. Functional antagonism has also putatively evolved in a subset of KAI2d proteins in parasitic plants through subfunctionalization. These proteins attenuate SL signaling, in opposition to other KAI2d SL receptors, by protecting SMAX1 (81). Like DLK2, these atypical KAI2d proteins have acquired mutations in key MAX2-interacting residues that may disrupt their ability to transduce SL signals (81).

### What is KL?

The identity of KL(s) remains a critical missing piece of the KAR/KL signaling puzzle. Although unknown, KL is anticipated to have several properties. First, the broad distribution of well-conserved eu-KAI2 proteins in land plants implies that KL has a widespread distribution that matches that of its receptors. Supporting this, a *KAI2* gene from *Selaginella moellendorffii* can partially rescue an *Arabidopsis kai2* mutant, suggesting that KAI2-activating molecules in *Arabidopsis* may be similar to those in *Selaginella* and other basally diverged land plants (22). Second, KL is likely to be a hydrolyzable molecule. SL perception by D14 relies heavily upon cleavage of a methyl butenolide “D-ring” that becomes covalently attached to a catalytic His residue (36, 37). Structurally similar molecules that are not cleavable show substantially reduced activity (82). A similar pattern is observed for molecules that can activate KAI2 (83, 84). The strict conservation of the catalytic triad Ser-His-Asp residues in land plant KAI2 proteins implies that they are functionally important (50). Indeed, mutation of these triad residues inactivates KAI2 proteins as it also does to D14 (22). Third, KL molecules may be structurally related to SL molecules but have different stereochemical configurations and a desmethyl butenolide D-ring. KAI2 responds to 2’*S*-configured enantiomers of SLs (which always have a 2’*R* configuration) and is particularly sensitive to desmethyl butenolide compounds compared to methyl butenolides (13, 24, 69, 85).

Four naturally occurring plant molecules have been found that are bound by some KAI2 proteins: 1) (–)-germacrene D, a volatile sesquiterpenoid molecule that activates PhKAI2ia during pistil development in petunia (*Petunia* × *hybrida*) flowers; 2) dehydrocostus lactone, a sesquiterpene that can bind covalently to KAI2 proteins from *A. thaliana* and a root parasitic plant, *Orobanche cumana*; 3) 2-phenethyl isothiocyanate, a glucosinolate breakdown product that binds and activates a KAI2d protein from the parasitic plant *Phelipanche ramosa*; and 4) zaxinone, a carotenoid-derived molecule that may be a competitive inhibitor of KL and SL perception by *A. thaliana* KAI2 and D14 (86–89). While certainly interesting, the limited distribution, chemical structures, different reactivities, and binding modes of (–)-germacrene D, dehydrocostus lactone, and 2-phenethyl isothiocyanate makes them unlikely to be “the” KL(s). The antagonistic effect of zaxinone on KAI2 excludes it from being a bioactive KL.

Given the diversity of molecules that are capable of binding and/or activating various KAI2 proteins, it will be unsurprising to find that more than one kind of KL is produced by plants. It is noteworthy that there is substantial, but far from complete overlap in the differential gene expression observed in *kuf1 dlk2* and *smax1 smxl2* mutants (1288 shared DEGs among 3579 *smax1 smxl2* DEGs). This may be because *smax1 smxl2* represents a severe, organism-wide SMAX1/SMXL2-deficient state that cannot be achieved even by highly active SCF^MAX2^-driven proteolysis, and/or perhaps due to hormetic effects on the expression of some genes (Fig. 3*C*). Alternatively, it may be that KUF1 and DLK2 regulate only a subset of KLs, with other metabolic control mechanisms waiting to be discovered.

The efforts of ourselves and many others to isolate KL(s) have likely been hampered by its low abundance and/or instability during extraction. The *kuf1 dlk2* mutant, which we have shown to be defective in two feedback mechanisms that restrict KAR/KL abundance, will putatively provide a rich source of KL(s) that may help to resolve these limitations.

## Materials and Methods

### Molecular Reagents.

The following compounds were prepared as described previously: KAR_1_ and KAR_2_ (2); dGR24 (24); YLG (71) and desmethyl-YLG (70). Individual enantiomers of GR24 were separated as previously described (85). 10 mM stocks were prepared in acetone and stored at −20 °C, and freshly diluted in aqueous solutions before use. For the root hair assay, KAR_2_ from (OlChemim, Czech Republic) was prepared in a 10 mM stock in 75% (*v/v*) methanol and stored at −20 °C. Genes were cloned into pGWBCitr (90) or pGWB502 (91) binary vectors for overexpression. See *SI* *Appendix* for details of plasmid construction.

### Plant Materials and Growth Conditions.

*A. thaliana* mutants *kai2* (*htl-3* allele), *kuf1-1*, *dlk2-1* (SALK_126829), *dlk2-3* (SALK_026193), *max2-1*, and *smax1-2 smxl2-1* are in the Col-0 background and were previously described (7, 17, 33, 43, 92). *Arabidopsis* seeds were surface-sterilized (5 min 70% (*v/v*) EtOH with 0.05% (*v/v*) Triton X-100, followed by 70% and 95% (*v/v*) EtOH washes, and air-drying) and grown on 0.5x Murashige-Skoog (MS) Medium with MES Buffer and Vitamins (Research Products International), pH 5.7, solidified with 0.8% (w/v) Bacto agar. *Arabidopsis* and *N. benthamiana* plants were grown under white light (MaxLite LED T8 4000K, ~110 µmol m^−2^ s^−1^) with a long-day photoperiod (16 h light/8 h dark) at 22 °C. Sunshine Mix #1 soil (Sun Gro Horticulture) was supplemented with Osmocote 14–14–14 fertilizer, Gnatrol WDG, and Marathon (imidacloprid). Primers for genotyping mutant alleles are found in *SI Appendix,* Table S4. Floral dip transformation of *A. thaliana* with *Agrobacterium tumefaciens* (GV3101 pMP90) was performed in 5% (*w/v*) sucrose with 0.025% (*v/v*) Silwet L-77 (93). All characterized transgenic lines were homozygous and carried a single copy transgene insertion in the T3 or higher generation.

### Figures and Statistical Analysis.

Figure creation and statistical analysis were performed with ggplot2 (94), GraphPad Prism 10, and Affinity Designer 2. Tests for significance were performed post hoc after one-way or two-way ANOVA as indicated. Boxplots use Tukey’s whiskers. Data that fall outside the whiskers are shown as individual points.

### Hypocotyl elongation assays.

Hypocotyl growth under red light was performed as described previously (43). Surface-sterilized seeds were plated on solid 0.5x MS media with 1 µM KAR_1_ or 0.01% (*v/v*) acetone as mock control, cold stratified for 3 d at 4 °C in darkness, then moved to a HiPoint DCI-700 LED Z4 growth chamber to grow at 21 °C under white light (150 µmol m^−2^ s^−1^) for 3 h, dark for 21 h, and red light (30 µmol m^−2^ s^−1^) for 4 d. Hypocotyl lengths of photographed seedlings were measured with ImageJ (NIH).

### Root Hair Assays.

Root hair development was assayed as described previously according to Villaécija-Aguilar et al. with some modifications (95). Images were taken with a Leica M165 FC microscope equipped with a Leica K5C camera. For quantification of root hair length, the length of all the root hairs visible in the correct plane was measured between 2 to 3 mm distance from the root tip using FIJI as described previously (95). The values obtained for one root were averaged to give the root hair length in that region for that root. For the quantification of root hair density, the number of visible root hairs between 2 to 3 mm distance away from the root tip was counted. This provided the density as root hair number per mm in that region per individual root.

### RNA-seq.

Total RNA was extracted from *Arabidopsis* seedlings grown under the same conditions as hypocotyl elongation assays. Four biological replicates (pools of seedlings from different plates) were collected, processed, and analyzed for each sample. Stranded libraries for RNA-seq were prepared using the NEB Ultra II Direction RNA Library kit with polyA mRNA isolatifon. Sequencing was performed on a NovaSeq S4 150PE flowcell. A minimum of 28 M uniquely mapped reads were obtained for each library (*SI Appendix,* Table S1). Reads were mapped with STAR (96). Differential expression analysis was performed on read count data in RStudio with the DESeq2 package (97). FCs of normalized read counts are reported for all DEGs in Dataset S1. Additional experimental details are described in *SI Appendix*.

### SMAX1 Reporter Assays in *A. thaliana*.

*A. thaliana* plants expressing *35S:SMAX1_D2_-LUC* (23) introgressed into different backgrounds were grown 9 d in a white 96-well plate (Perkin Elmer OptiPlate 96) containing 200 µL 0.5x MS agar medium then sprayed with 2 mM D-luciferin (Gold Biotechnology) and incubated 3 h before further treatment to equilibrate. 10 µM KAR_1_, 10 µM KAR_2_, 10 µM dGR24, or 0.1% (*v/v*) acetone solvent control were then sprayed along with 2 mM D-luciferin. Luciferase signal was measured using a CLARIOstar plate reader (BMG Labtech) under controlled 21 °C temperature. For normalization of untreated seedlings, total leaf and cotyledon surface area was measured in ImageJ. For degradation analysis, the relative luciferase activity of each plant at each time point was calculated as a percentage of activity at time zero (cps_tn_ * 100)/cps_t0_.

### Transient Expression Assays in *N. benthamiana*.

Transient expression assays were performed as described with slight modifications (23). *A. tumefaciens* strain GV3101 was used for transformation. Strains carrying *pRATIO1212-SMAX1* or *pRATIO1212-SMXL7* reporters also expressed the p19 protein. *Agrobacterium* strains carrying constructs as indicated were coinfiltrated into 3-wk-old leaves of *N. benthamiana plants*. Leaf discs were excised 3 d postinfiltration, moved to a black 96-well polystyrene plate (PE OptiPlate 96, PerkinElmer) abaxial side up, and measured in CLARIOstar plate reader (BMG Labtech) in plate mode (slow kinetics) with the following settings: spiral scan option; scan diameter (mm), 5; and number of flashes per well per cycle, 36. Optimal settings for fluorescence measurements of the mScarlet-I reporter (ex. 560–10 nm, em. 595–10 nm) and Venus reference (ex. 497–15 nm, em. 540–20 nm) proteins were described previously (60). Gain for each fluorophore was adjusted in each experiment based upon samples with the strongest signal intensity. The mScarlet-I/Venus fluorescence intensity ratios were calculated after subtracting background fluorescence signals measured in leaf discs transformed with p19 alone. Chemical treatments were performed on leaf discs postexcision by floating in solution at room temperature before fluorescence analysis at indicated time points.

### Confocal Fluorescence Microscopy.

Seedlings of NES/NLS-fused DLK2-mT2 transgenic lines were grown on 0.5× MS medium supplemented with 0.8% agar for 7 d. Whole roots were dipped in propidium iodide (PI; 100 µg/mL) solution for 20 min. After staining, roots were mounted on glass slides in water and imaged with an LSM880 Elyra confocal microscope (Zeiss). For PI, fluorescence excitation was at 561 nm and emission was collected at 570 to 718 nm. For mTurquoise2, fluorescence excitation was at 458 nm and emission was collected at 490 to 570 nm.

### Split-Luciferase Complementation Assay.

*KAI2* and *kai2^S95A^* CDS were cloned into pCAMBIA1300-cLUC (98). *SMAX1^∆RGKT^* and *SMXL2* CDS were cloned into pCAMBIA1300-nLUC using NEBuilder. The nLUC and cLUC fusion plasmids was transiently coexpressed with *35S*:*GFP*, *35S*:*DLK2,* or *35S*:*dlk2^S102A^* via agroinfiltration in leaves of 3-wk-old wild-type *N. benthamiana* plants as described previously, with OD_600_ 0.5 for each *A. tumefaciens* culture (99). An *Agrobacterium* strain containing *35S*:*mCherry* was used as a transformation control. After 3 d, leaf discs were excised and immersed in 150 μL of 2 mM D-luciferin solution with 10 µM KAR_1_ or dGR24 in a 96-well white plate (PE OptiPlate 96) and kept in the dark for 5 min before measurement on a CLARIOstar plate reader (BMG Labtech). Luminescence was measured using the emission filter 580 nm (80 nm bandwidth) and normalized to fluorescence of mCherry.

### Hydrolysis Assays.

Molecular cloning, protein expression, and protein purification of SUMO-tagged AtDLK2, BtDLK2 [Genbank MG783333.1; (72)], BtDLK2^S102A^, and AtKAI2 (AT4G37470) were performed as described previously (24, 100). See *SI Appendix* for details.

## Supplementary Material

Appendix 01 (PDF)

Dataset S01 (XLSX)

Dataset S02 (TXT)

## Data Availability

Study data are included in the article and/or *SI Appendix*. Sequencing data generated in this study have been deposited in the NCBI BioProject database under accession number PRJNA1320694 (101).

## References

[1] G. R. Flematti, E. L. Ghisalberti, K. W. Dixon, R. D. Trengove, A compound from smoke that promotes seed germination. Science **305**, 977 (2004). 15247439 10.1126/science.1099944

[2] G. R. Flematti , Preparation of 2H-furo[2, 3-c]pyran-2-one derivatives and evaluation of their germination-promoting activity. J. Agric. Food Chem. **55**, 2189–2194 (2007). 17316021 10.1021/jf0633241

[3] D. C. Nelson, G. R. Flematti, E. L. Ghisalberti, K. W. Dixon, S. M. Smith, Regulation of seed germination and seedling growth by chemical signals from burning vegetation. Annu. Rev. Plant Biol. **63**, 107–130 (2012). 22404467 10.1146/annurev-arplant-042811-105545

[4] D. C. Nelson , Karrikins discovered in smoke trigger *Arabidopsis* seed germination by a mechanism requiring gibberellic acid synthesis and light. Plant Physiol. **149**, 863–873 (2009). 19074625 10.1104/pp.108.131516 PMC2633839

[5] D. C. Nelson , Karrikins enhance light responses during germination and seedling development in *Arabidopsis thaliana*. Proc. Natl. Acad. Sci. U.S.A. **107**, 7095–7100 (2010). 20351290 10.1073/pnas.0911635107 PMC2872431

[6] J. A. Villaécija-Aguilar , SMAX1/SMXL2 regulate root and root hair development downstream of KAI2-mediated signalling in *Arabidopsis*. PLoS Genet. **15**, e1008327 (2019). 31465451 10.1371/journal.pgen.1008327 PMC6738646

[7] M. T. Waters , Specialisation within the DWARF14 protein family confers distinct responses to karrikins and strigolactones in *Arabidopsis*. Development **139**, 1285–1295 (2012). 22357928 10.1242/dev.074567

[8] C. Gutjahr , Rice perception of symbiotic arbuscular mycorrhizal fungi requires the karrikin receptor complex. Science **350**, 1521–1524 (2015). 26680197 10.1126/science.aac9715

[9] J. Zheng , Karrikin signaling acts parallel to and additively with strigolactone signaling to regulate rice mesocotyl elongation in darkness. Plant Cell **32**, 2780–2805 (2020). 32665307 10.1105/tpc.20.00123 PMC7474294

[10] J. Choi , The negative regulator SMAX1 controls mycorrhizal symbiosis and strigolactone biosynthesis in rice. Nat. Commun. **11**, 2114 (2020). 32355217 10.1038/s41467-020-16021-1 PMC7193599

[11] X.-R. Li , Nutrient regulation of lipochitooligosaccharide recognition in plants via NSP1 and NSP2. Nat. Commun. **13**, 6421 (2022). 36307431 10.1038/s41467-022-33908-3 PMC9616857

[12] S. Carbonnel , Lotus japonicus karrikin receptors display divergent ligand-binding specificities and organ-dependent redundancy. PLoS Genet. **16**, e1009249 (2020). 33370251 10.1371/journal.pgen.1009249 PMC7808659

[13] Y. Meng , Karrikin insensitive2 regulates leaf development, root system architecture and arbuscular-mycorrhizal symbiosis in *Brachypodium distachyon*. Plant J. **109**, 1559–1574 (2022). 34953105 10.1111/tpj.15651

[14] M. T. Waters, D. C. Nelson, Karrikin perception and signalling. New Phytol. **237**, 1525–1541 (2022). 36333982 10.1111/nph.18598

[15] Y. Guo, Z. Zheng, J. J. La Clair, J. Chory, J. P. Noel, Smoke-derived karrikin perception by the α/β-hydrolase KAI2 from *Arabidopsis*. Proc. Natl. Acad. Sci. U.S.A. **110**, 8284–8289 (2013). 23613584 10.1073/pnas.1306265110 PMC3657771

[16] M. Kagiyama , Structures of D14 and D14L in the strigolactone and karrikin signaling pathways. Genes Cells **18**, 147–160 (2013). 23301669 10.1111/gtc.12025

[17] S. Toh, D. Holbrook-Smith, M. E. Stokes, Y. Tsuchiya, P. McCourt, Detection of parasitic plant suicide germination compounds using a high-throughput *Arabidopsis* HTL/KAI2 strigolactone perception system. Chem. Biol. **21**, 988–998 (2014). 25126711 10.1016/j.chembiol.2014.07.005

[18] Y. Xu , Structural basis of unique ligand specificity of KAI2-like protein from parasitic weed Striga hermonthica. Sci. Rep. **6**, 31386 (2016). 27507097 10.1038/srep31386 PMC4979206

[19] Y. Xu , Structural analysis of HTL and D14 proteins reveals the basis for ligand selectivity in Striga. Nat. Commun. **9**, 3947 (2018). 30258184 10.1038/s41467-018-06452-2 PMC6158167

[20] I. Lee , A missense allele of KARRIKIN-INSENSITIVE2 impairs ligand-binding and downstream signaling in *Arabidopsis thaliana*. J. Exp. Bot. **69**, 3609–3623 (2018). 29722815 10.1093/jxb/ery164 PMC6022639

[21] M. Bürger , Structural basis of karrikin and non-natural strigolactone perception in *Physcomitrella patens*. Cell Rep. **26**, 855–865.e5 (2019). 30673608 10.1016/j.celrep.2019.01.003 PMC7233462

[22] M. T. Waters , A *Selaginella moellendorffii* ortholog of KARRIKIN INSENSITIVE2 functions in *Arabidopsis* development but cannot mediate responses to karrikins or strigolactones. Plant Cell **27**, 1925–1944 (2015). 26175507 10.1105/tpc.15.00146 PMC4531350

[23] A. Khosla , Structure-function analysis of SMAX1 reveals domains that mediate its karrikin-induced proteolysis and interaction with the receptor KAI2. Plant Cell **32**, 2639–2659 (2020). 32434855 10.1105/tpc.19.00752 PMC7401016

[24] J. Yao , Desmethyl butenolides are optimal ligands for karrikin receptor proteins. New Phytol. **230**, 1003–1016 (2021). 33474738 10.1111/nph.17224

[25] X.-D. Sun, M. Ni, Hyposensitive to light, an alpha/beta fold protein, acts downstream of elongated hypocotyl 5 to regulate seedling de-etiolation. Mol. Plant **4**, 116–126 (2011). 20864454 10.1093/mp/ssq055

[26] W. Li , The karrikin receptor KAI2 promotes drought resistance in *Arabidopsis thaliana*. PLoS Genet. **13**, e1007076 (2017). 29131815 10.1371/journal.pgen.1007076 PMC5703579

[27] C. E. Conn, D. C. Nelson, Evidence that KARRIKIN-INSENSITIVE2 (KAI2) receptors may perceive an unknown signal that is not Karrikin or Strigolactone. Front. Plant Sci. **6**, 1219 (2015). 26779242 10.3389/fpls.2015.01219 PMC4705300

[28] Y. Mizuno , Major components of the KARRIKIN INSENSITIVE2-dependent signaling pathway are conserved in the liverwort Marchantia polymorpha. Plant Cell **33**, 2395–2411 (2021). 33839776 10.1093/plcell/koab106 PMC8364241

[29] M. Lopez-Obando , The Physcomitrium (Physcomitrella) patens PpKAI2L receptors for strigolactones and related compounds function via MAX2-dependent and -independent pathways. Plant Cell **33**, 3487–3512 (2021). 34459915 10.1093/plcell/koab217 PMC8662777

[30] M. A. Blázquez, D. C. Nelson, D. Weijers, Evolution of plant hormone response pathways. Annu. Rev. Plant Biol. **71**, 327–353 (2020). 32017604 10.1146/annurev-arplant-050718-100309

[31] L. Wang , Strigolactone and karrikin signaling pathways elicit ubiquitination and proteolysis of SMXL2 to regulate hypocotyl elongation in *Arabidopsis*. Plant Cell **32**, 2251–2270 (2020). 32358074 10.1105/tpc.20.00140 PMC7346549

[32] J. P. Stanga, S. M. Smith, W. R. Briggs, D. C. Nelson, Suppressor of more axillary growth2 1 controls seed germination and seedling development in *Arabidopsis*. Plant Physiol. **163**, 318–330 (2013). 23893171 10.1104/pp.113.221259 PMC3762653

[33] J. P. Stanga, N. Morffy, D. C. Nelson, Functional redundancy in the control of seedling growth by the karrikin signaling pathway. Planta **243**, 1397–1406 (2016). 26754282 10.1007/s00425-015-2458-2 PMC7676495

[34] S. H. Chang, W. George, D. C. Nelson, Transcriptional regulation of development by SMAX1-LIKE proteins, targets of strigolactone and karrikin/KAI2 ligand signaling. J. Exp. Bot. **76**, 1888–1906 (2025). 39869020 10.1093/jxb/eraf027

[35] C. Hamiaux , DAD2 is an α/β hydrolase likely to be involved in the perception of the plant branching hormone, strigolactone. Curr. Biol. **22**, 2032–2036 (2012). 22959345 10.1016/j.cub.2012.08.007

[36] R. Yao , DWARF14 is a non-canonical hormone receptor for strigolactone. Nature **536**, 469–473 (2016). 27479325 10.1038/nature19073

[37] A. de Saint Germain , An histidine covalent receptor and butenolide complex mediates strigolactone perception. Nat. Chem. Biol. **12**, 787–794 (2016). 27479744 10.1038/nchembio.2147 PMC5030144

[38] Y. Seto , Strigolactone perception and deactivation by a hydrolase receptor DWARF14. Nat. Commun. **10**, 191 (2019). 30643123 10.1038/s41467-018-08124-7 PMC6331613

[39] L. Jiang , DWARF 53 acts as a repressor of strigolactone signalling in rice. Nature **504**, 401–405 (2013). 24336200 10.1038/nature12870 PMC5802366

[40] F. Zhou , D14-SCF(D3)-dependent degradation of D53 regulates strigolactone signalling. Nature **504**, 406–410 (2013). 24336215 10.1038/nature12878 PMC4096652

[41] L. Wang , Strigolactone signaling in *Arabidopsis* regulates shoot development by targeting D53-like SMXL repressor proteins for ubiquitination and degradation. Plant Cell **27**, 3128–3142 (2015). 26546446 10.1105/tpc.15.00605 PMC4682305

[42] Q. Li , The strigolactone receptor D14 targets SMAX1 for degradation in response to GR24 treatment and osmotic stress. Plant Commun. **3**, 100303 (2022). 35529949 10.1016/j.xplc.2022.100303 PMC9073322

[43] C. Sepulveda , KARRIKIN UP-REGULATED F-BOX 1 (KUF1) imposes negative feedback regulation of karrikin and KAI2 ligand metabolism in *Arabidopsis thaliana*. Proc. Natl. Acad. Sci. U.S.A. **119**, e2112820119 (2022). 35254909 10.1073/pnas.2112820119 PMC8931227

[44] H. Tian , Karrikin upregulated F-box 1 negatively regulates drought tolerance in *Arabidopsis*. Plant Physiol. **190**, 2671–2687 (2022). 35822606 10.1093/plphys/kiac336 PMC9706471

[45] M. Lopez-Obando , Physcomitrella patens MAX2 characterization suggests an ancient role for this F-box protein in photomorphogenesis rather than strigolactone signalling. New Phytol. **219**, 743–756 (2018). 29781136 10.1111/nph.15214

[46] S. C. Kerr , Hormonal regulation of the BRC1-dependent strigolactone transcriptome involved in shoot branching responses. bioRxiv [Preprint] (2020), 10.1101/2020.03.19.999581 (Accessed 20 March 2020).

[47] A. Végh , Comprehensive analysis of DWARF14-LIKE2 (DLK2) reveals its functional divergence from Strigolactone-related paralogs. Front. Plant Sci. **8**, 1641 (2017). 28970845 10.3389/fpls.2017.01641 PMC5609103

[48] T. Sisaphaithong , Localized expression of the Dwarf14-like2a gene in rice roots on infection of arbuscular mycorrhizal fungus and hydrolysis of rac-GR24 by the encoded protein. Plant Signal. Behav. **16**, 2009998 (2021). 34904518 10.1080/15592324.2021.2009998 PMC9208777

[49] M. Bürger , Crystal structure of *Arabidopsis* DWARF14-LIKE2 (DLK2) reveals a distinct substrate binding pocket architecture. Plant Direct **6**, e446 (2022). 36172078 10.1002/pld3.446 PMC9470386

[50] R. Bythell-Douglas , Evolution of strigolactone receptors by gradual neo-functionalization of KAI2 paralogues. BMC Biol. **15**, 52 (2017). 28662667 10.1186/s12915-017-0397-z PMC5490202

[51] A. I. Vancea , Mechanism of cooperative strigolactone perception by the MAX2 ubiquitin ligase-receptor-substrate complex. Nat. Commun. **16**, 10291 (2025). 41271672 10.1038/s41467-025-65205-0 PMC12639087

[52] L.-H. Zhao , Destabilization of strigolactone receptor DWARF14 by binding of ligand and E3-ligase signaling effector DWARF3. Cell Res. **25**, 1219–1236 (2015). 26470846 10.1038/cr.2015.122 PMC4650425

[53] F. Chevalier , Strigolactone promotes degradation of DWARF14, an α/β hydrolase essential for strigolactone signaling in *Arabidopsis*. Plant Cell **26**, 1134–1150 (2014). 24610723 10.1105/tpc.114.122903 PMC4001374

[54] E. S. Martín-Fontecha, F. Cardinale, M. Bürger, C. Prandi, P. Cubas, Novel mechanisms of strigolactone-induced DWARF14 degradation in *Arabidopsis thaliana*. J. Exp. Bot. **75**, 22 (2024). 10.1093/jxb/erae365 PMC11630080 39196982

[55] M. Bunsick , HTL/KAI2 signalling substitutes for light to control plant germination. bioRxiv [Preprint] (2022), https://www.biorxiv.org/content/10.1101/2022.03.30.486460v1 (Accessed 20 March 2022). 10.1371/journal.pgen.1011447 PMC11527322 39432524

[56] T. Ho-Plágaro , DLK2 regulates arbuscule hyphal branching during arbuscular mycorrhizal symbiosis. New Phytol. **229**, 548–562 (2021). 32966595 10.1111/nph.16938

[57] M. Ramos-Alvelo , The SlDLK2 receptor, involved in the control of arbuscular mycorrhizal symbiosis, regulates hormonal balance in roots. Front. Microbiol. **15**, 1472449 (2024). 39723137 10.3389/fmicb.2024.1472449 PMC11668738

[58] M. Du , Biphasic control of cell expansion by auxin coordinates etiolated seedling development. Sci. Adv. **8**, eabj1570 (2022). 35020423 10.1126/sciadv.abj1570 PMC8754305

[59] S. Carbonnel , The karrikin signaling regulator SMAX1 controls Lotus japonicus root and root hair development by suppressing ethylene biosynthesis. Proc. Natl. Acad. Sci. U.S.A. **117**, 21757–21765 (2020). 32817510 10.1073/pnas.2006111117 PMC7474694

[60] A. Khosla, C. Rodriguez-Furlan, S. Kapoor, J. M. Van Norman, D. C. Nelson, A series of dual-reporter vectors for ratiometric analysis of protein abundance in plants. Plant Direct **4**, e00231 (2020). 32582876 10.1002/pld3.231 PMC7306620

[61] P. Stirnberg, I. J. Furner, H. M. Ottoline Leyser, MAX2 participates in an SCF complex which acts locally at the node to suppress shoot branching. Plant J. **50**, 80–94 (2007). 17346265 10.1111/j.1365-313X.2007.03032.x

[62] H. Shen, P. Luong, E. Huq, The F-box protein MAX2 functions as a positive regulator of photomorphogenesis in *Arabidopsis*. Plant Physiol. **145**, 1471–1483 (2007). 17951458 10.1104/pp.107.107227 PMC2151697

[63] I. Soundappan , SMAX1-LIKE/D53 family members enable distinct MAX2-dependent responses to strigolactones and karrikins in *Arabidopsis*. Plant Cell **27**, 3143–3159 (2015). 26546447 10.1105/tpc.15.00562 PMC4682302

[64] Y. Liang, S. Ward, P. Li, T. Bennett, O. Leyser, SMAX1-LIKE7 signals from the nucleus to regulate shoot development in *Arabidopsis* via partially EAR motif-independent mechanisms. Plant Cell **28**, 1581–1601 (2016). 27317673 10.1105/tpc.16.00286 PMC4981136

[65] S. R. Cutler, D. W. Ehrhardt, J. S. Griffitts, C. R. Somerville, Random GFP∷ cDNA fusions enable visualization of subcellular structures in cells of *Arabidopsis* at a high frequency. Proc. Natl. Acad. Sci. U.S.A. **97**, 3718–3723 (2000). 10737809 10.1073/pnas.97.7.3718 PMC16306

[66] J. Goedhart , Structure-guided evolution of cyan fluorescent proteins towards a quantum yield of 93%. Nat. Commun. **3**, 751 (2012). 22434194 10.1038/ncomms1738 PMC3316892

[67] D. Haasen, C. Kohler, G. Neuhaus, T. Merkle, Nuclear export of proteins in plants: AtXPO1 is the export receptor for leucine-rich nuclear export signals in *Arabidopsis thaliana*. Plant J. **20**, 695–705 (1999). 10652141 10.1046/j.1365-313x.1999.00644.x

[68] S. A. Chevalier , Presence of a functional but dispensable nuclear export signal in the HTLV-2 Tax protein. Retrovirology **2**, 70 (2005). 16285885 10.1186/1742-4690-2-70 PMC1308865

[69] S. Okabe , Desmethyl type germinone, a specific agonist for the HTL/KAI2 receptor, induces the *Arabidopsis* seed germination in a gibberellin-independent manner. Biochem. Biophys. Res. Commun. **649**, 110–117 (2023). 36764113 10.1016/j.bbrc.2023.01.086

[70] J. Yao , An allelic series at the KARRIKIN INSENSITIVE 2 locus of *Arabidopsis thaliana* decouples ligand hydrolysis and receptor degradation from downstream signalling. Plant J. **96**, 75–89 (2018). 29982999 10.1111/tpj.14017

[71] Y. Tsuchiya , Probing strigolactone receptors in Striga hermonthica with fluorescence. Science **349**, 864–868 (2015). 26293962 10.1126/science.aab3831

[72] Y. K. Sun , Divergent receptor proteins confer responses to different karrikins in two ephemeral weeds. Nat. Commun. **11**, 1264 (2020). 32152287 10.1038/s41467-020-14991-w PMC7062792

[73] J.-M. Davière, P. Achard, A pivotal role of DELLAs in regulating multiple hormone signals. Mol. Plant **9**, 10–20 (2016). 26415696 10.1016/j.molp.2015.09.011

[74] H. Kameoka , Dienelactone hydrolase like protein1 negatively regulates the KAI2-ligand pathway in *Marchantia polymorpha*. Curr. Biol. **33**, 3505–3513.e5 (2023). 37480853 10.1016/j.cub.2023.06.083

[75] E. Xu , Catabolism of strigolactones by a carboxylesterase. Nat. Plants **7**, 1495–1504 (2021). 34764442 10.1038/s41477-021-01011-y

[76] M. Palayam , Structural insights into strigolactone catabolism by carboxylesterases reveal a conserved conformational regulation. Nat. Commun. **15**, 6500 (2024). 39090154 10.1038/s41467-024-50928-3 PMC11294565

[77] A. J. Tuckey , Structural and functional characterisation of a reconstructed ancestral strigolactone receptor. bioRxiv [Preprint] (2025), 10.1101/2025.08.03.668049 (Accessed 8 April 2025). PMC13575467 42740481

[78] C. E. Conn , Convergent evolution of strigolactone perception enabled host detection in parasitic plants. Science **349**, 540–543 (2015). 26228149 10.1126/science.aab1140

[79] D. C. Nelson, The mechanism of host-induced germination in root parasitic plants. Plant Physiol. **185**, 1353–1373 (2021). 33793958 10.1093/plphys/kiab043 PMC8133615

[80] A. Arellano-Saab , Three mutations repurpose a plant karrikin receptor to a strigolactone receptor. Proc. Natl. Acad. Sci. U.S.A. **118**, e2103175118 (2021). 34301902 10.1073/pnas.2103175118 PMC8325247

[81] A. R. F. White, A. Kane, S. Ogawa, K. Shirasu, D. C. Nelson, Dominant-negative KAI2d paralogs putatively attenuate strigolactone responses in root parasitic plants. Plant Cell Physiol. **65**, 1969–1982 (2024). 39275795 10.1093/pcp/pcae106

[82] J. Takeuchi , Rationally designed strigolactone analogs as antagonists of the D14 receptor. Plant Cell Physiol. **59**, 1545–1554 (2018). 29727000 10.1093/pcp/pcy087

[83] R. Kushihara , Structural requirements of KAI2 ligands for activation of signal transduction. Proc. Natl. Acad. Sci. U.S.A. **122**, e2414779122 (2025). 39977316 10.1073/pnas.2414779122 PMC11874195

[84] D. Uraguchi , A femtomolar-range suicide germination stimulant for the parasitic plant Striga hermonthica. Science **362**, 1301–1305 (2018). 30545887 10.1126/science.aau5445

[85] A. Scaffidi , Strigolactone hormones and their stereoisomers signal through two related receptor proteins to induce different physiological responses in *Arabidopsis*. Plant Physiol. **165**, 1221–1232 (2014). 24808100 10.1104/pp.114.240036 PMC4081333

[86] S. A. Stirling , Volatile communication in plants relies on a KAI2-mediated signaling pathway. Science **383**, 1318–1325 (2024). 38513014 10.1126/science.adl4685

[87] J. C. Moreno ., Arabidopsis response to the apocarotenoid zaxinone involves interference with strigolactone signaling via binding to DWARF14. *Nat. Commun.* **16**, 8789 (2025). 10.1038/s41467-025-63845-w PMC12491641 41038837

[88] S. Han , Naturally occurring dehydrocostus lactone covalently binds to KARRIKIN INSENSITIVE 2 by dual Serine modifications in Orobanche cumana and *Arabidopsis*. J. Agric. Food Chem. **72**, 19920–19930 (2024). 39213540 10.1021/acs.jafc.4c06359

[89] A. de Saint Germain , A Phelipanche ramosa KAI2 protein perceives strigolactones and isothiocyanates enzymatically. Plant Commun. **2**, 100166 (2021). 34746757 10.1016/j.xplc.2021.100166 PMC8553955

[90] S. H. Chang, W. J. George, D. C. Nelson, An N-terminal domain specifies developmental control by the SMAX1-LIKE family of transcriptional regulators in *Arabidopsis thaliana*. Proc. Natl. Acad. Sci. U.S.A. **122**, e2412793122 (2025). 40493196 10.1073/pnas.2412793122 PMC12184505

[91] T. Nakagawa , Improved gateway binary vectors: High-performance vectors for creation of fusion constructs in transgenic analysis of plants. Biosci. Biotechnol. Biochem. **71**, 2095–2100 (2007). 17690442 10.1271/bbb.70216

[92] P. Stirnberg, K. van De Sande, H. M. O. Leyser, MAX1 and MAX2 control shoot lateral branching in *Arabidopsis*. Development **129**, 1131–1141 (2002). 11874909 10.1242/dev.129.5.1131

[93] S. J. Clough, A. F. Bent, Floral dip: A simplified method for *Agrobacterium*-mediated transformation of *Arabidopsis thaliana*. Plant J. **16**, 735–743 (1998). 10069079 10.1046/j.1365-313x.1998.00343.x

[94] H. Wickham, ggplot2: Elegant Graphics for Data Analysis (Springer, 2016).

[95] J. A. Villaécija-Aguilar, S. Struk, S. Goormachtig, C. Gutjahr, Bioassays for the effects of strigolactones and other small molecules on root and root hair development. Methods Mol. Biol. **2309**, 129–142 (2021). 34028684 10.1007/978-1-0716-1429-7_11

[96] A. Dobin , STAR: Ultrafast universal RNA-seq aligner. Bioinformatics **29**, 15–21 (2013). 23104886 10.1093/bioinformatics/bts635 PMC3530905

[97] M. I. Love, W. Huber, S. Anders, Moderated estimation of fold change and dispersion for RNA-seq data with DESeq2. Genome Biol. **15**, 550 (2014). 25516281 10.1186/s13059-014-0550-8 PMC4302049

[98] H. Chen , Firefly luciferase complementation imaging assay for protein-protein interactions in plants. Plant Physiol. **146**, 368–376 (2008). 18065554 10.1104/pp.107.111740 PMC2245818

[99] Q. Li , SMXL5 attenuates strigolactone signaling in *Arabidopsis thaliana* by inhibiting SMXL7 degradation. Mol. Plant **17**, 631–647 (2024). 38475994 10.1016/j.molp.2024.03.006

[100] M. T. Waters, A. Scaffidi, G. Flematti, S. M. Smith, Substrate-induced degradation of the α/β-fold hydrolase KARRIKIN INSENSITIVE2 requires a functional catalytic triad but is independent of MAX2. Mol. Plant **8**, 814–817 (2015). 25698586 10.1016/j.molp.2014.12.020

[101] D. C. Nelson, Feedback regulation of karrikin signaling by KUF1 and DLK2 in Arabidopsis thaliana. NCBI BioProject. https://www.ncbi.nlm.nih.gov/bioproject/1320694. Deposited 11 October 2025.

