# Supplementary material for: Negative feedback regulation of karrikin signaling in Arabidopsis thaliana by an antagonistic paralog of karrikin receptors
Source: Proc Natl Acad Sci U S A. 2026 Sep 8;123(37):e2525145123. doi: 10.1073/pnas.2525145123 (PMC13578778; doi:10.1073/pnas.2525145123)
Supplement: Supplementary file 1 [file pnas.2525145123.sapp.pdf]

PNAS

Supporting Information for

Negative feedback regulation of karrikin signaling in *Arabidopsis thaliana* by an antagonistic paralog of karrikin receptors

Qingtian Li[1,2]✝, Sun Hyun Chang[1]✝, Andrew Tuckey[3], Claudia Sepulveda[1], Kartikye Varshney[4], Dan Li[2], Caroline Gutjahr[4], Mark T. Waters[3], David C. Nelson[1]

Corresponding Author: David C. Nelson
Email: david.nelson@ucr.edu

This PDF file includes:

- Supporting text
- Figures S1 to S12
- Tables S1 to S4
- Legends for Dataset S1

Other supporting materials for this manuscript include the following:

- Dataset S1
- Dataset S2

Supporting Information Text

MATERIALS AND METHODS

Hydrolysis assays

Coding sequences were codon-optimised and synthesized (Genscript) for cloning into pE-SUMO-Amp expression vector (LifeSensors). pE-SUMO-BtDLK2^{S102A} was created by inverse PCR with mutagenic primers using the WT BtDLK2 clone as a template. Hydrolysis assays were performed as described previously, with protein concentration adjusted so that each 10 µL reaction contained 4 µg protein. Hydrolysis assays were performed with technical triplicates in 96-well black assay plates (Greiner 655900). Proteins were diluted to 100 µg/mL in hydrolysis buffer (100 mM HEPES, 150 mM NaCl, pH 7). Profluorescent substrates (Yoshimulactone Green (YLG) or desmethyl Yoshimulactone Green (dYLG)) were serially diluted in DMSO to form 1000× stocks, which were then diluted in hydrolysis buffer to 1.11× reaction conditions immediately prior to dispensing on the assay plate. Final substrate concentrations ranged from 0 to 20 µM. With the 96-well plate on ice, 10 µL protein (4 µg) was dispensed per well, followed by 90 µL of substrate. The plate was loaded immediately into a plate reader at 25 °C (Biotek Synergy HTX), shaking the plate orbitally for 20 s, then measuring fluorescence (excitation/emission of 485/520 nm) every minute, for 30 minutes. Blank-subtracted fluorescence values (from wells without protein) were analysed in GraphPad Prism using nonlinear regression with the Michaelis-Menten model. The initial hydrolysis rate was defined as the change in blank-subtracted fluorescence units over the first 8 minutes.

Plasmid construction

The *Δkuf1* variant was made by Bgl II restriction digestion which dropped out 517-nt of the *KUF1* (AT1G31350.1) coding sequence (CDS). The CDS of *DLK2* (AT3G24420) and *dlk2^{S102A}* were synthesized (Twist Bioscience) and cloned into pDONR221A by Gateway BP reaction (Invitrogen). *DLK2* and *dlk2^{S102A}* were subsequently transferred into pGWBCitr to generate *35S*:*DLK2* and *35S*:*dlk2^{S102A}*, respectively. To generate constructs that fused DLK2 with NES or NLS, a DNA sequence containing the 315 bp of DLK2 from 3' end of CDS with no stop codon, following the CDS of mTurquoise2 with no stop codon, an SpeI site, N7 NLS with a stop codon, a BamHI site, NES2 with a stop codon, a NdeI site, AtRanBP1b NES with a stop codon, a NcoI site, a stop codon and a overhang sequence on pENTR3C (5'-AATTCGCGGCCGCACTCG-3') was synthesized. The 504 bp of DLK2 from start codon was amplified by PCR with primer pairs of DLK2-3C-F and DLK2-NLS/NES-R, and subsequently assembled with BamHI-EcoRI-digested pENTR3C and synthesized DNA using NEBuilder HiFi DNA Assembly Master Mix to construct a DLK2-mTurquoise2-N7 NLS-NES2-AtRanBP1b NES entry clone. Then the entry clone was digested with SpeI and BamHI, and repaired with an overlapping oligo (5'-ATGGATGAGCTTTATAAAACTAGTGGATCCAAGAGGCTTGAGGAACTT-3') using HiFi DNA Assembly Master Mix to generate the DLK2-mTurquoise2-NES2 fusion; digested with SpeI and NdeI, and repaired with an overlapping oligo (5'-ATGGATGAGCTTTATAAAACTAGTCATATGGATACTGCGGGCTTATTA-3') to generate the DLK2-mTurquoise2-AtRanBP1b NES fusion; digested with BamHI and NcoI, and repaired with an overlapping oligo (5'-GACCAAGAGGAAGAGTAGGGATCCCCATGGTAAAATTCGCGGCCGCAC-3') to generate the DLK2-mTurquoise2-N7 NLS fusion; digested with SpeI and NcoI, and repaired with an overlapping oligo (5'-ATGGATGAGCTTTATAAAACTAGTCCATGGTAAAATTCGCGGCCGCAC-3') to generate the DLK2-mTurquoise2 fusion. Sequences of oligonucleotide primers used for construct synthesis are provided in *SI Appendix,* Table S4.

Fusions were transferred into pGWB502 binary vector by Gateway LR reaction (Invitrogen) to generate *35S*:*DLK2-mTurquoise2-NES2*, *35S*:*DLK2-mTurquoise2-AtRanBP1b NES*, *35S*:*DLK2-mTurquoise2-N7 NLS*, and *35S*:*DLK2-mTurquoise2*. CDS for *BtDLK2, MpKAI2B, OsD14L2, OsD14L3, and SlDLK2* were synthesized (Twist Bioscience) and cloned into pDONR221 using Gateway BP clonase. The entry clones of *AtDLK2, BtDLK2, MpKAI2B, OsD14L2, OsD14L3, and SlDLK* were introduced into pGWB502 binary vector through Gateway LR reaction to generate *35S::AtDLK2, 35S::BtDLK2, 35S::MpKAI2B, 35S::OsD14L2, 35S::OsD14L3, and 35S::SlDLK2*. Synthesized sequences are provided in *SI Appendix,* Dataset S2.

RNA-seq

Surface-sterilized seeds were stratified in the dark for 3 d at 4 °C, then moved to a HiPoint DCI-700 LED Z4 growth chamber to grow at 21 °C under white light (150 μmol m^{-2} s^{-1}) for 3 h, dark for 21 h, and red light (30 μmol m^{-2} s^{-1}) for 4 d. Seedlings were harvested and immediately frozen in liquid N_2 before storage at -80°C. Tissue was ground with a power drill and plastic pestle in a centrifuge tube in liquid N_2. RNA was extracted from 50 to 100 mg of frozen tissue with Spectrum Plant Total RNA Kit (Sigma) including on-column DNase I digestion (Sigma). RNA concentrations were measured by Qubit RNA Broad-Range Assay kit and Fluorometer (Invitrogen). RNA integrity was tested by the 2100 Bioanalyzer (Agilent). Four biological replicates (pools of seedlings from different plates) were collected, processed, and analyzed for each sample. Stranded libraries for RNA-seq were prepared by UC Riverside Genomics Core from ~500 ng total RNA per sample using NEB Ultra II Direction RNA Library kit (E7765) with polyA mRNA isolation (E7490) with the following modifications: a 0.8× bead clean was performed after 2nd strand synthesis, adaptor was diluted 1:30, a 0.7× bead clean was performed after ligation, amplification was performed for 13 cycles, and a dual bead size selection was performed at the final clean up. Sequencing was performed on a NovaSeq S4 150PE flowcell. Raw paired-end read data are available at NCBI SRA (Bioproject ID PRJNA1320694). Reads were mapped onto the Athaliana_447_TAIR10 genome sequence with Araport11_GTF_genes_transposons.201606.gtf gene annotation using STAR 2.7.10a (94) with the following options (--sjdbOverhang 149; --alignIntronMax 15000; --quantMode TranscriptomeSAM GeneCounts). A minimum of 28 M uniquely mapped reads were obtained for each library (*SI Appendix,* Table S1). Differential expression analysis was performed on read count data in RStudio with the DESeq2 package (95). Fold-changes of normalized read counts are reported for all DEGs in *SI Appendix,* Dataset S1.

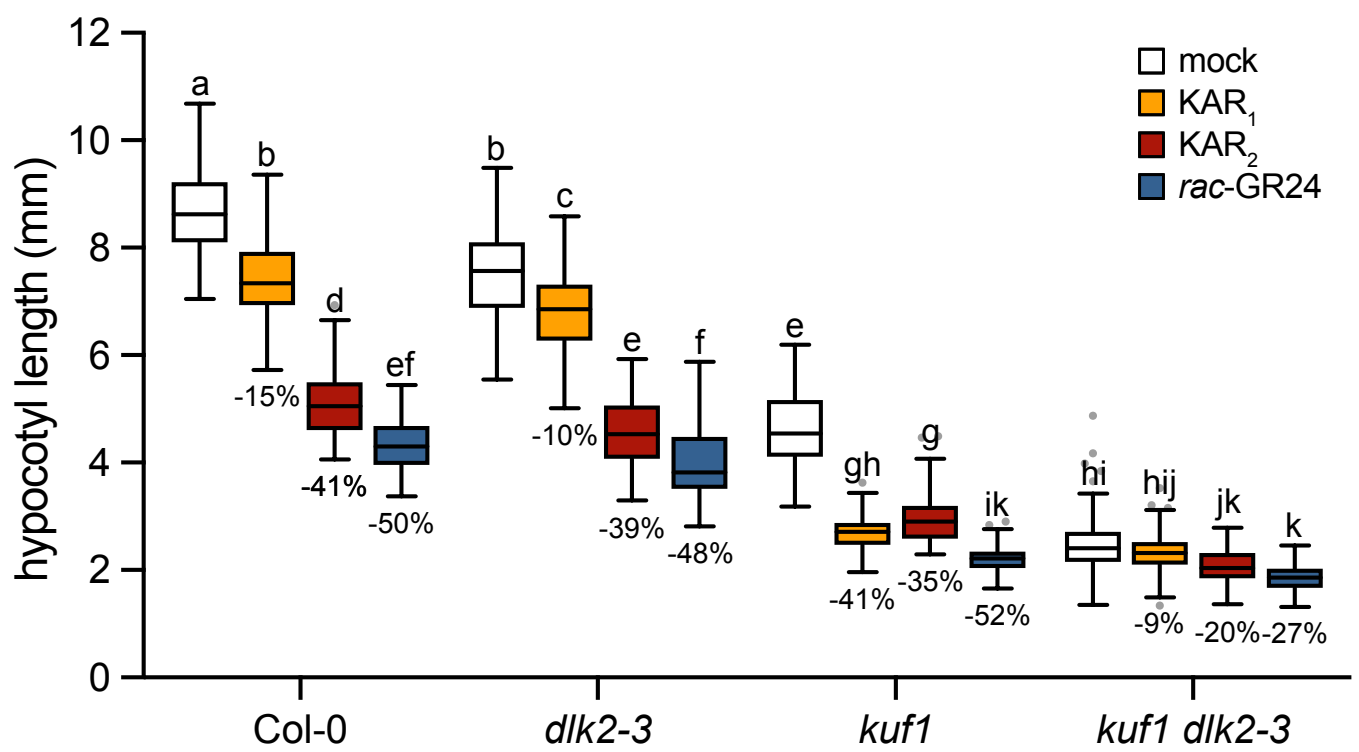

Figure S1. *dlk2* enhances *kuf1* but responds normally to KARs and *rac*-GR24
Hypocotyl length of 5-d-old *Arabidopsis thaliana* seedlings grown in red light on 0.5xMS media with solvent control (0.1% (*v/v*) acetone), 1 µM KAR_1, KAR_2, or *rac*-GR24. Percentages indicate degree of hypocotyl growth inhibition relative to mock control for each genotype. $p<0.01$, two-way ANOVA with post hoc Tukey's multiple comparisons test; n=60. Box plots shown with Tukey's whiskers. Letters indicate statistical groups.

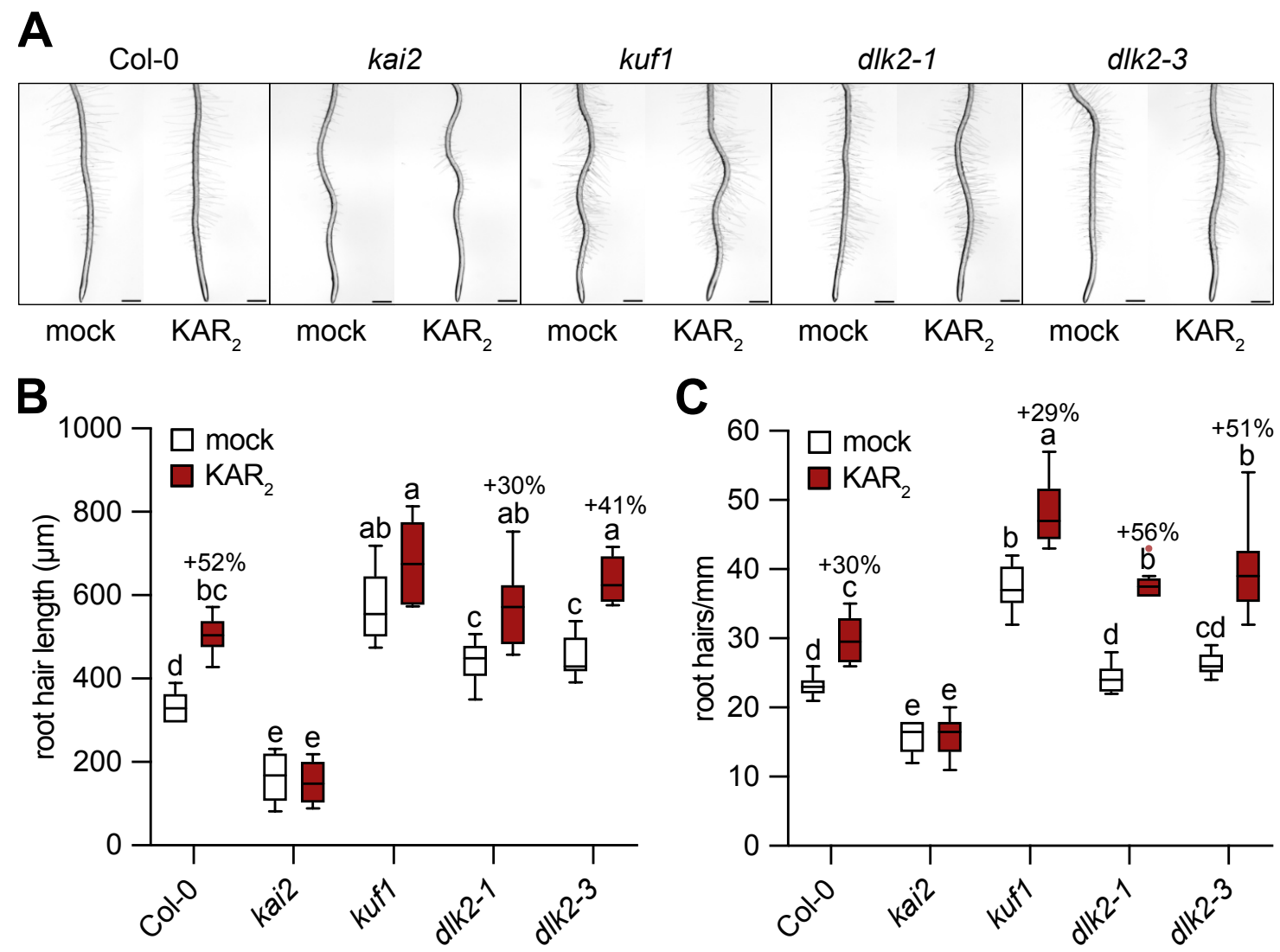

Figure S2. Root hair density is more enhanced by KAR_2 in *dlk2* seedlings

A) Representative images of root tips of 5-d-old *Arabidopsis thaliana* seedlings with either 1 µM KAR_2 or solvent (0.0075% (*v/v*) methanol) treatment. Scale bar, 500 µm. B) Root hair length and C) root hair density of 5-d-old seedlings. For each seedling, the mean length or density of all root hairs visible between 2 to 3 mm above the root tip was determined. Percentages indicate degree of root hair density increase by KAR_2. $p<0.05$, two-way ANOVA with post hoc Tukey's multiple comparisons test; n=8. Box plots shown with Tukey's whiskers. Letters indicate statistical groups.

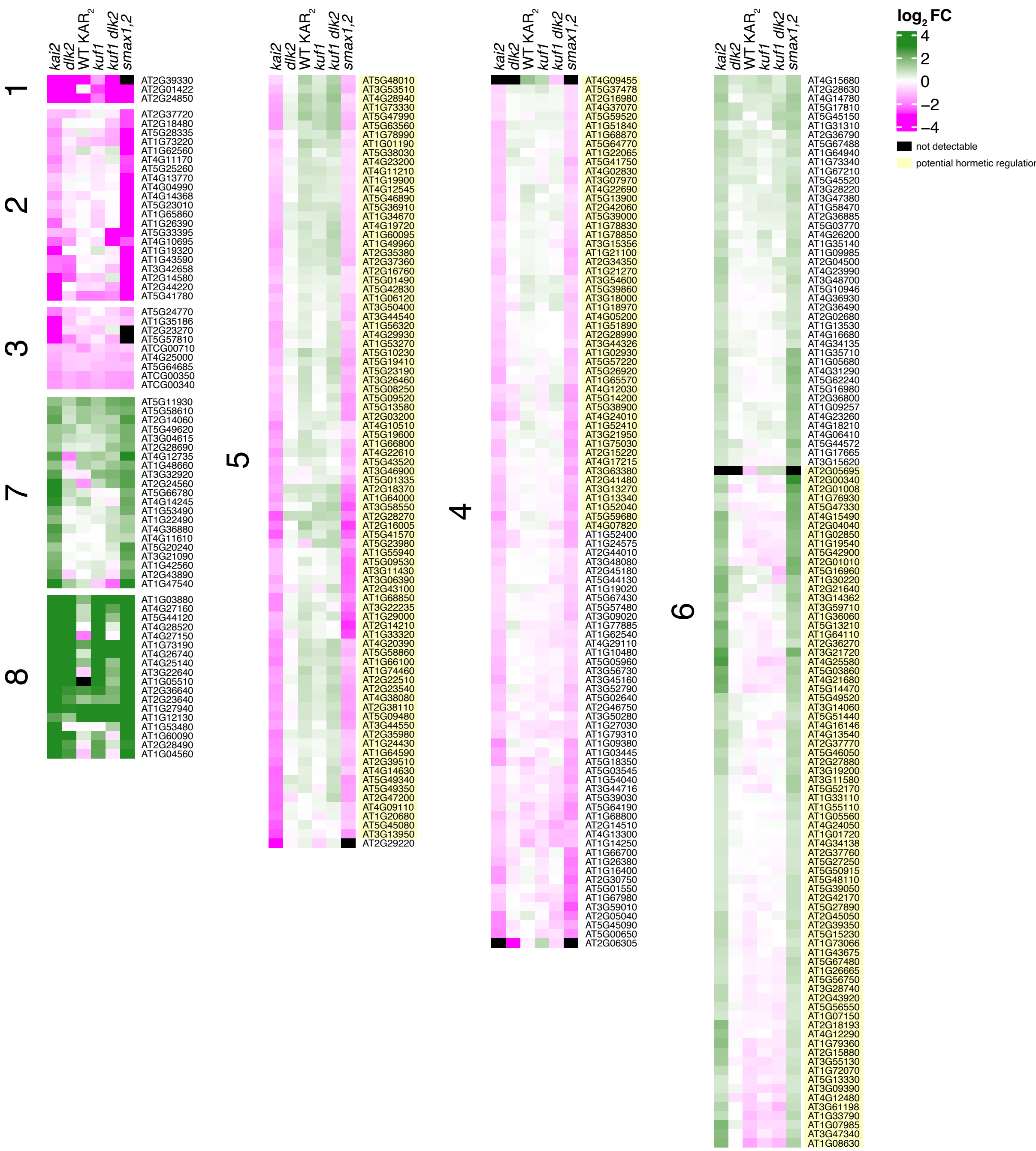

Figure S3. Heatmap of DEGs with similar directionality in *kai2* and *smax1 smxl2*
371 DEGs with similar directions of expression change in *kai2* and *smax1 smxl2*. Heatmap was generated with ComplexHeatmap package in R with eight gene clusters. Fold-changes in expression are relative to untreated WT (Col-0) and were $\log_2$-transformed. Genes that appeared to show a different direction of expression change in *kai2* and *smax1 smxl2* relative to KAR_2-treated WT, *kuf1*, and *kuf1 dlk2* are highlighted in yellow to indicate those that may be under hormetic regulation by KAR/KL signaling.

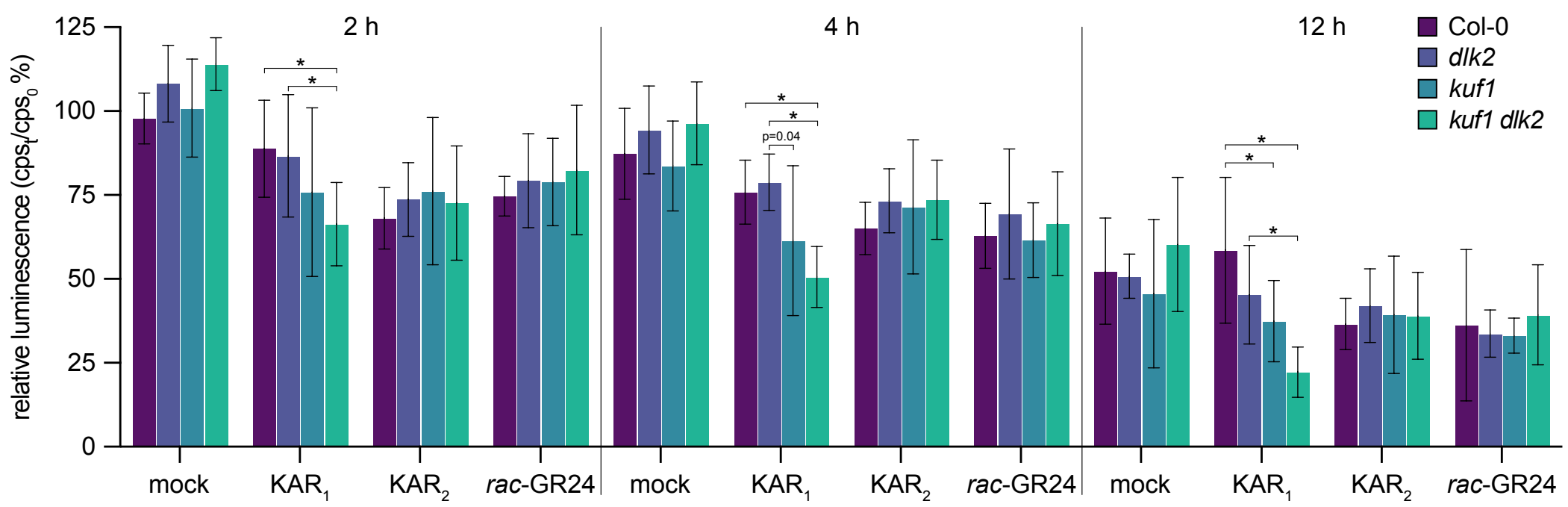

Figure S4. SMAX1 reporter is degraded more rapidly after KAR_1 treatment in *kuf1 dlk2*
Relative luminescence per seedling after 2, 4, and 12 h of treatment with 10 μM KAR_1, KAR_2, or *rac*-GR24 compared to luminescence at time 0. The $SMAX1_{D2}$-LUC translational reporter was expressed in 9-d-old seedlings grown in 16 h white light:8 h dark photoperiod. mean +/- s.d; *, $p<0.01$, one-way ANOVA with post hoc Tukey's multiple comparisons test comparing all genotypes within each time point x treatment group; n=10.

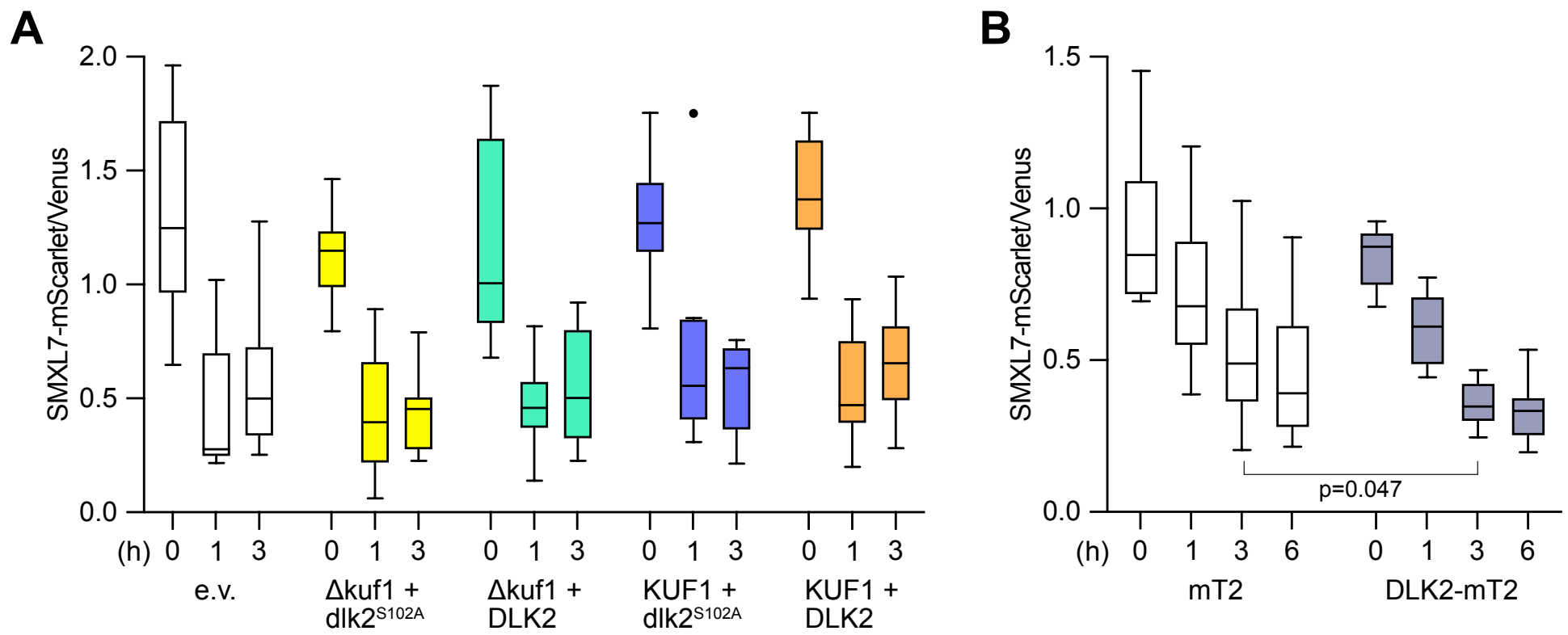

Figure S5. *DLK2* coexpression does not affect SMXL7 stability

Relative fluorescence of SMXL7-mScarlet to Venus in *N. benthamiana* leaves co-infiltrated with constructs that overexpress empty vector (e.v., pCAMBIA1300-cLUC), *KUF1*, *DLK2*, Δ*kuf1*, or *dlk2*^{S102A}. *DLK2* was fused with *cLUC*. Δ*kuf1* produces the N-terminal 46 aa of KUF1 only due to a deletion. *dlk2*^{S102A} has a mutation in a catalytic triad residue. Leaf discs were treated with 10 μM GR24^{5DS} and assayed after 1 and 3 h. $p<0.05$, two-way ANOVA with post hoc Dunnett's multiple comparisons test of discs with co-expressed KUF1/DLK2 genes to e.v. negative control at each time point; n=10-12 leaf discs. B) As in A), but with co-expression of mTurquoise2 (mT2) or DLK2-mT2 fusion with SMXL7 ratiometric reporter. Leaf discs were treated with 10 μM GR24^{5DS} and assayed after 1, 3, and 6 h. $p<0.01$, two-way ANOVA with post hoc Šídák's multiple comparisons test of DLK2-mT2 to mT2 at each time point; n=14 leaf discs. Box plots shown with Tukey's whiskers.

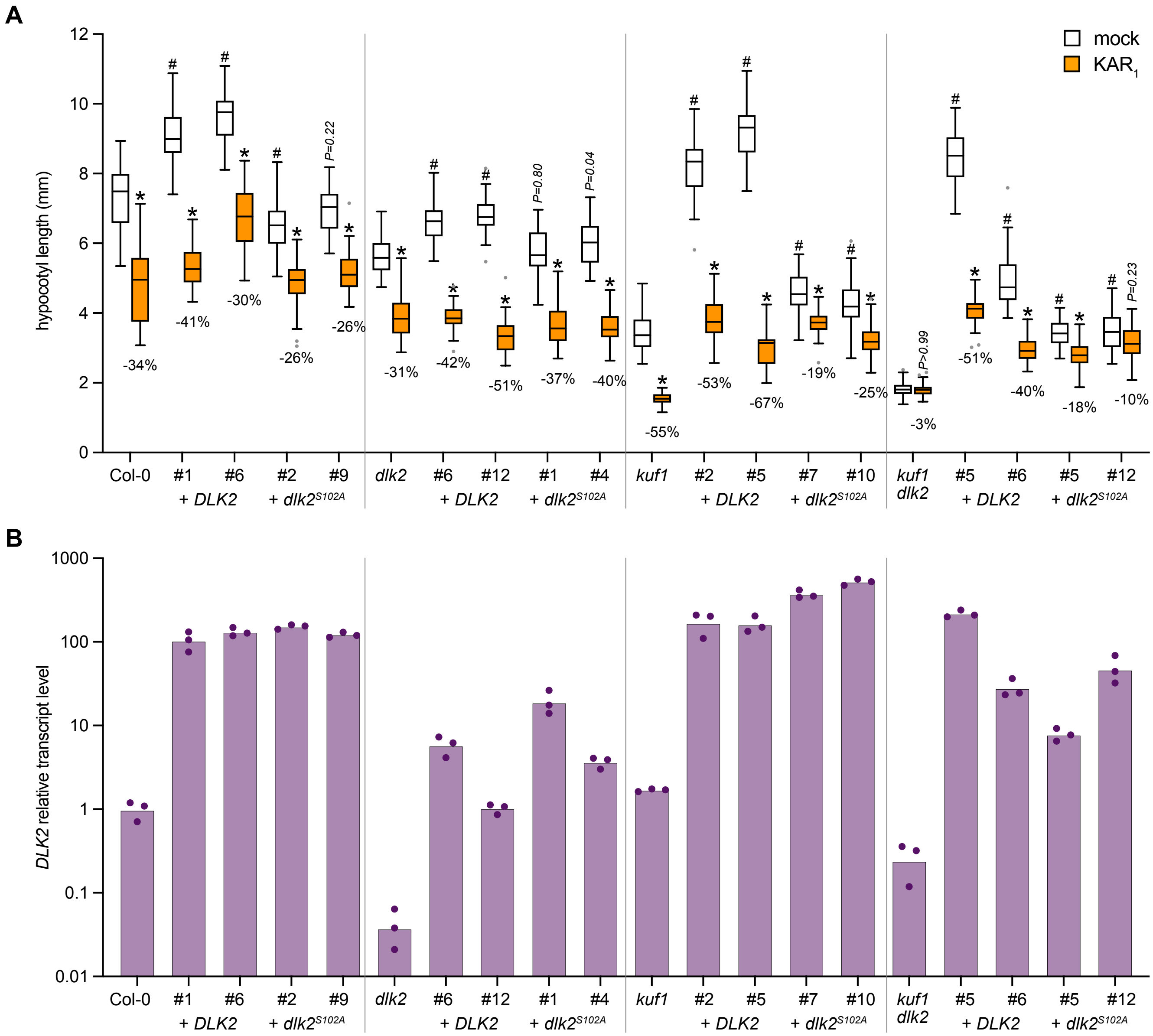

Figure S6. Effects of *DLK2* and *dlk2*^{S102A} overexpression on hypocotyl elongation

A) Hypocotyl length of 5-d-old *Arabidopsis thaliana* seedlings grown in red light on 0.5xMS media with or without 1 μM KAR_1. Percentages indicate degree of hypocotyl growth inhibition by KAR_1. *DLK2* and *dlk2*^{S102A} transgenes were expressed under control of the *35S* promoter in Col-0, *dlk2-1*, *kuf1-1*, or *kuf1-1 dlk2-1* backgrounds. Data for two independent homozygous lines per transgene are shown. Vertical gray lines separate groups by genetic background. #, p<0.01, Dunnett's T3 multiple comparisons test of mock-treated transgenic versus mock-treated non-transgenic WT or mutant seedlings of the same genetic background; n=32-45 seedlings. *, p<0.01, Šídák's multiple comparisons test of KAR_1-treated versus mock-treated seedlings of the same genotype; n=32-45 seedlings. Box plots shown with Tukey's whiskers. Data for *Col-0*, *kuf1*, *kuf1 35S:DLK2*, and *kuf1 35S:dlk2*^{S102A} are duplicated in Figure 4D. B) RT-qPCR analysis of *DLK2* transcript abundance relative to *CACS* reference in lines shown in A). Values have been re-scaled relative to Col-0. n=3 biological replicates. Note that y-axis is log-scale.

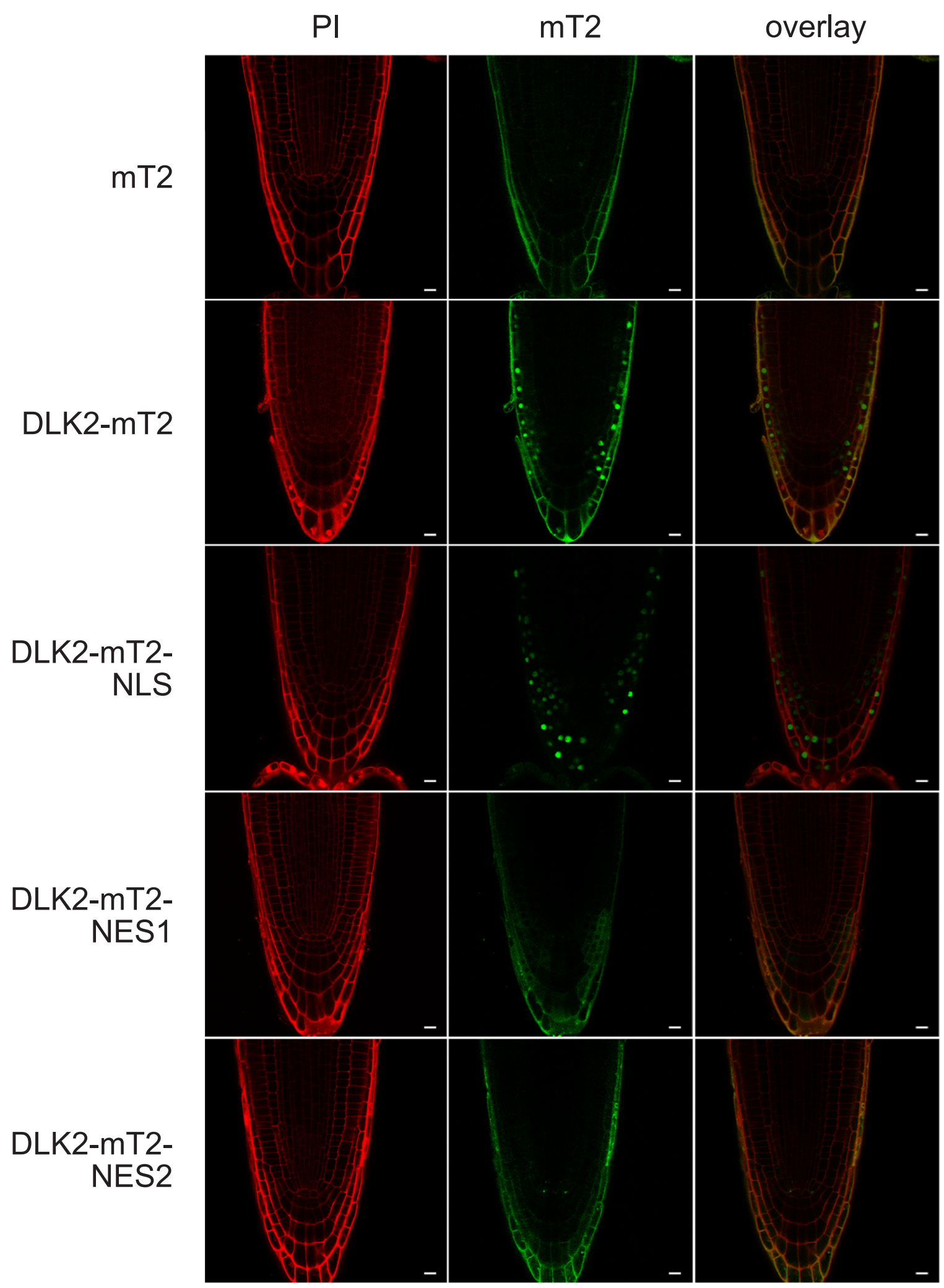

Figure S7. Confocal fluorescence microscopy of DLK2 fusion proteins in *Arabidopsis*
Subcellular localization of DLK2 fusions to mTurquoise2 and a C-terminal nuclear localization sequence (NLS) or nuclear exclusion sequence (NES) in transgenic *Arabidopsis thaliana dlk2 kuf1* roots (lines are shown in Fig. 5*D*). Fluorescence was detected by confocal laser scanning microscopy. Representative images are shown. PI, propidium iodide (red); mT2, mTurquoise2 (green); overlay, merged PI and mT2 signals. Scale bar, 10 μm.

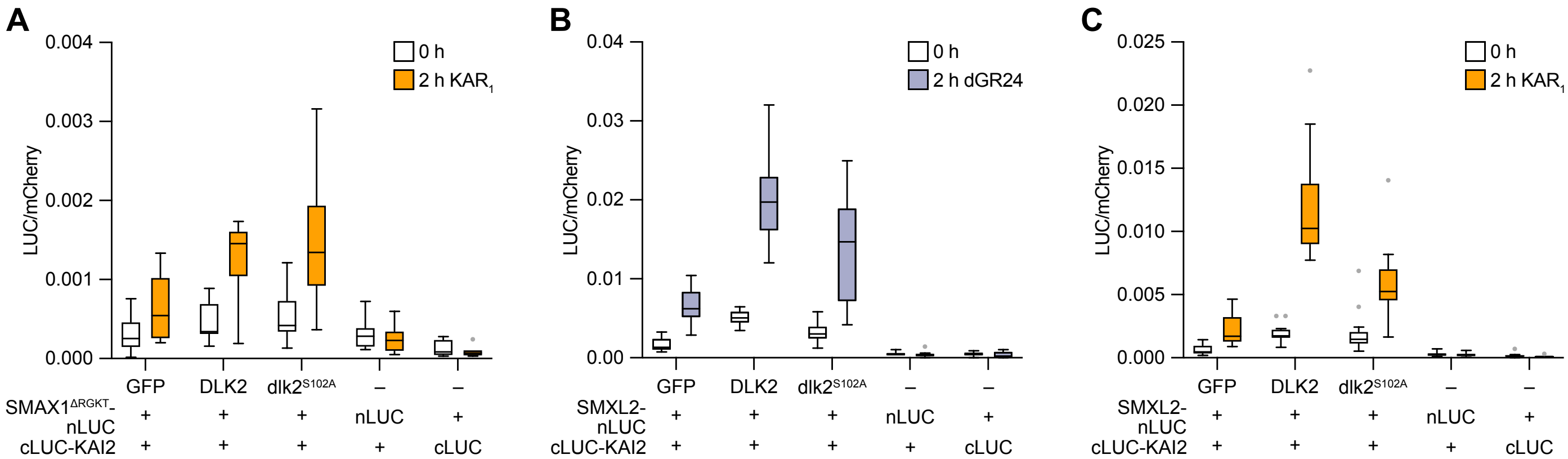

Figure S8. *DLK2* coexpression does not block KAI2-SMAX1 interactions
Split-luciferase assays for *DLK2* effect on A) KAI2-SMAX1$^{\Delta RGKT}$ and B-C) KAI2-SMXL2 protein-protein interactions in *N. benthamiana* leaves. *GFP*, *DLK2*, or *dlk2*^{S102A} were coexpressed. Luminescence was normalized to fluorescence from an mCherry reporter used as a transformation control. Leaf discs were measured before and after 2 h treatment with 10 µM KAR_1 or dGR24. n=8-15 leaf discs. Box plots shown with Tukey's whiskers.

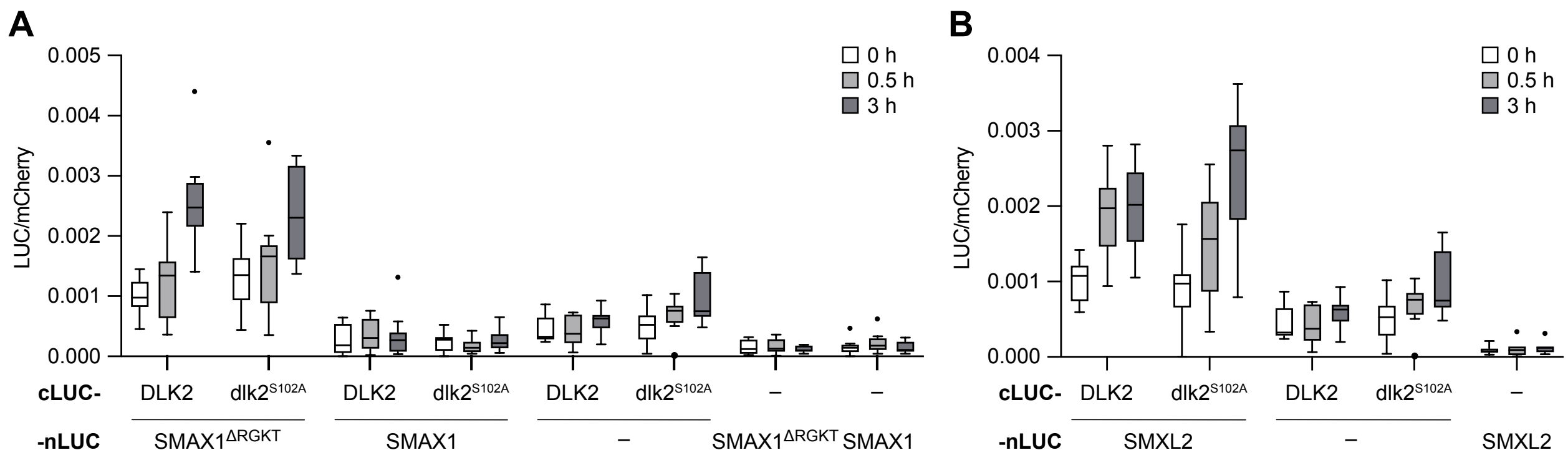

Figure S9. DLK2 and dlk2^{S102A} may interact similarly with SMAX1$^{\Delta RGKT}$ and SMXL2
Split-luciferase assays for DLK2 and dlk2^{S102A} protein-protein interactions with SMAX1$^{\Delta RGKT}$ and SMAX1 in *N. benthamiana* leaves. Luminescence was normalized to fluorescence from an mCherry reporter used as a transformation control. Leaf discs were measured before and after 0.5 and 3 h treatment with 10 µM dGR24. n=8-12 leaf discs. B) As for A) but testing for interactions with SMXL2. n=8-14 leaf discs. Box plots shown with Tukey's whiskers.

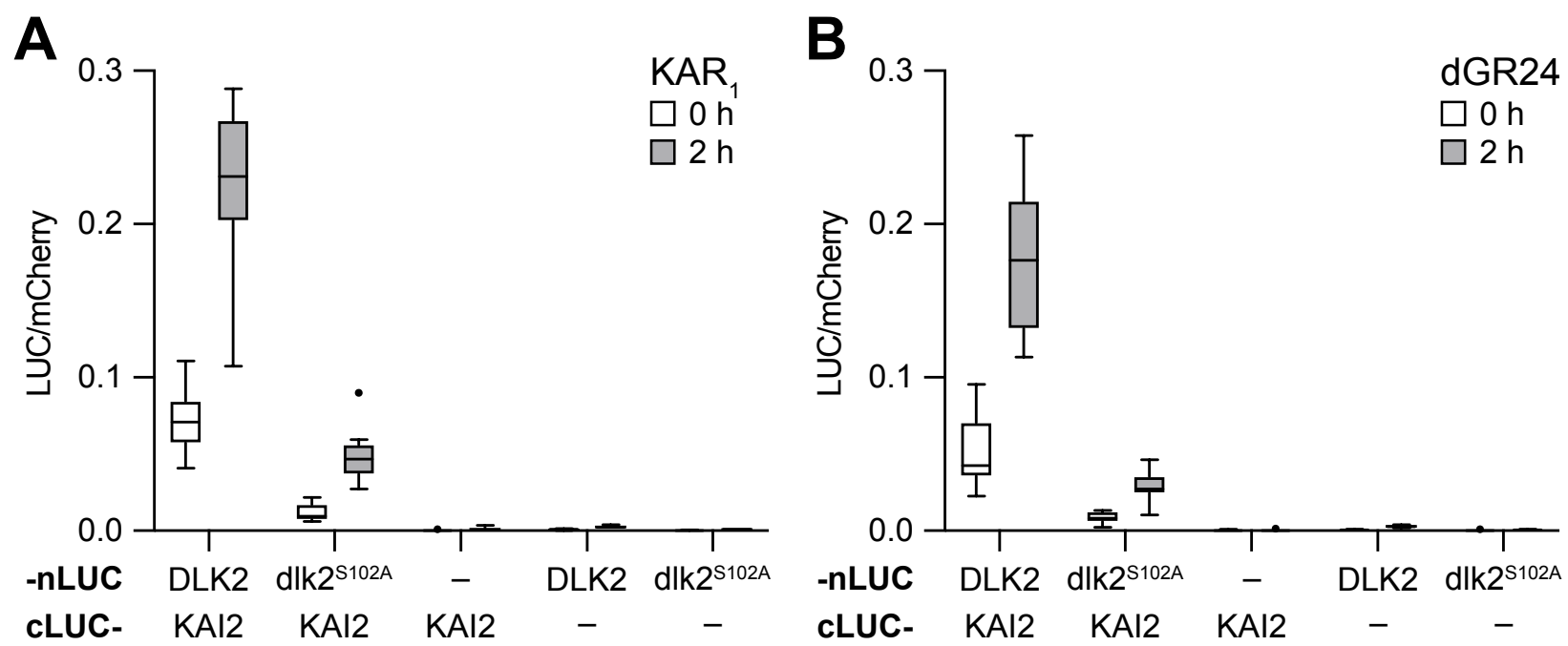

Figure S10. DLK2 shows evidence of interaction with KAI2

Split-luciferase assays for DLK2 and dlk2^{S102A} protein-protein interactions with KAI2 in *N. benthamiana* leaves. Luminescence was normalized to fluorescence from an mCherry reporter used as a transformation control. Leaf discs were measured before and after 2 h treatment with A) 10 µM KAR_1 or B) 10 µM dGR24. n=8-16 leaf discs.

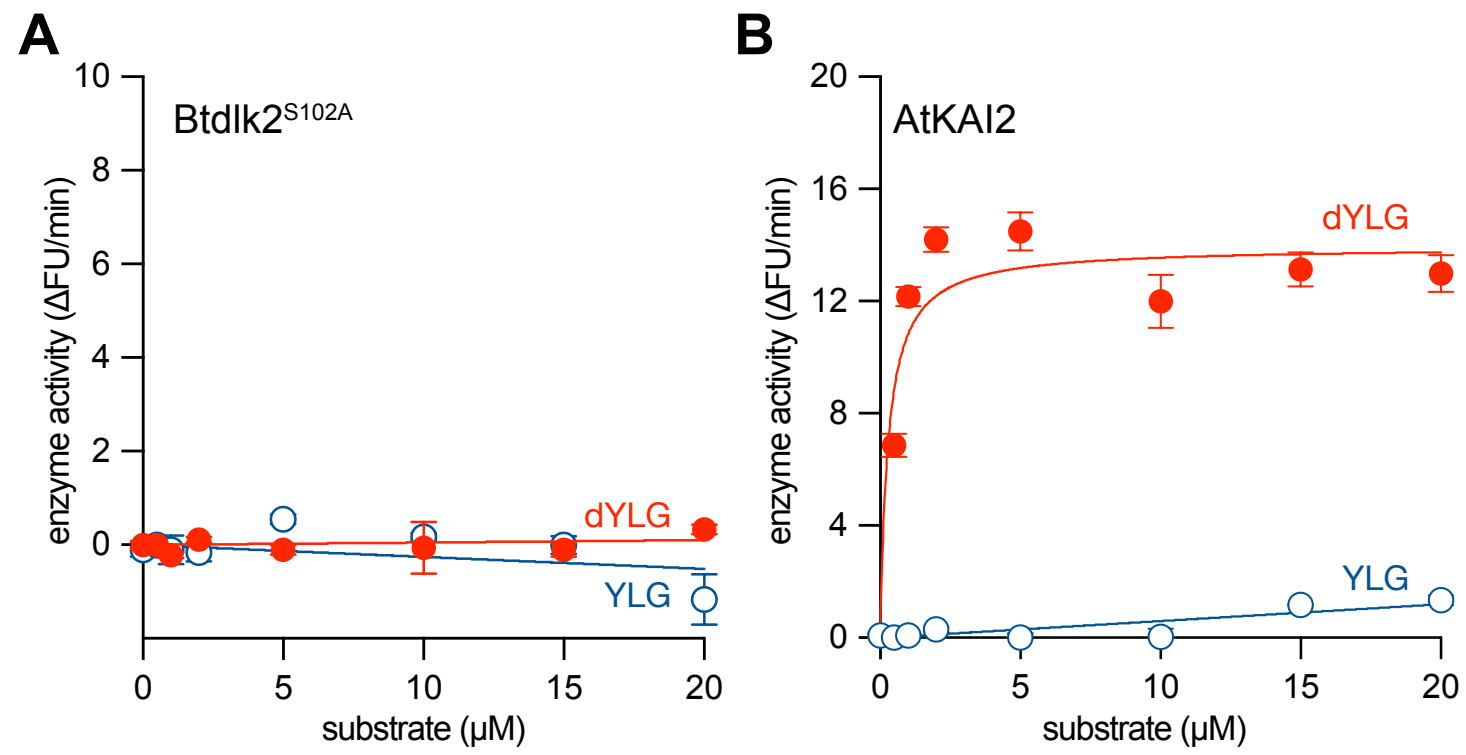

Figure S11. The catalytic triad Ser residue is necessary for BtDLK2 hydrolytic activity
In vitro hydrolysis of profluorescent reporter molecules yoshimulactone green (YLG) and desmethyl yoshimulactone green (dYLG) by 4 µg of A) SUMO-Btdlk2^{S102A} or B) SUMO-AtKAI2 in 100 mM HEPES, 150 mM NaCl, pH 7. Blank-subtracted mean ± s.e.m. of n=3 technical replicates.

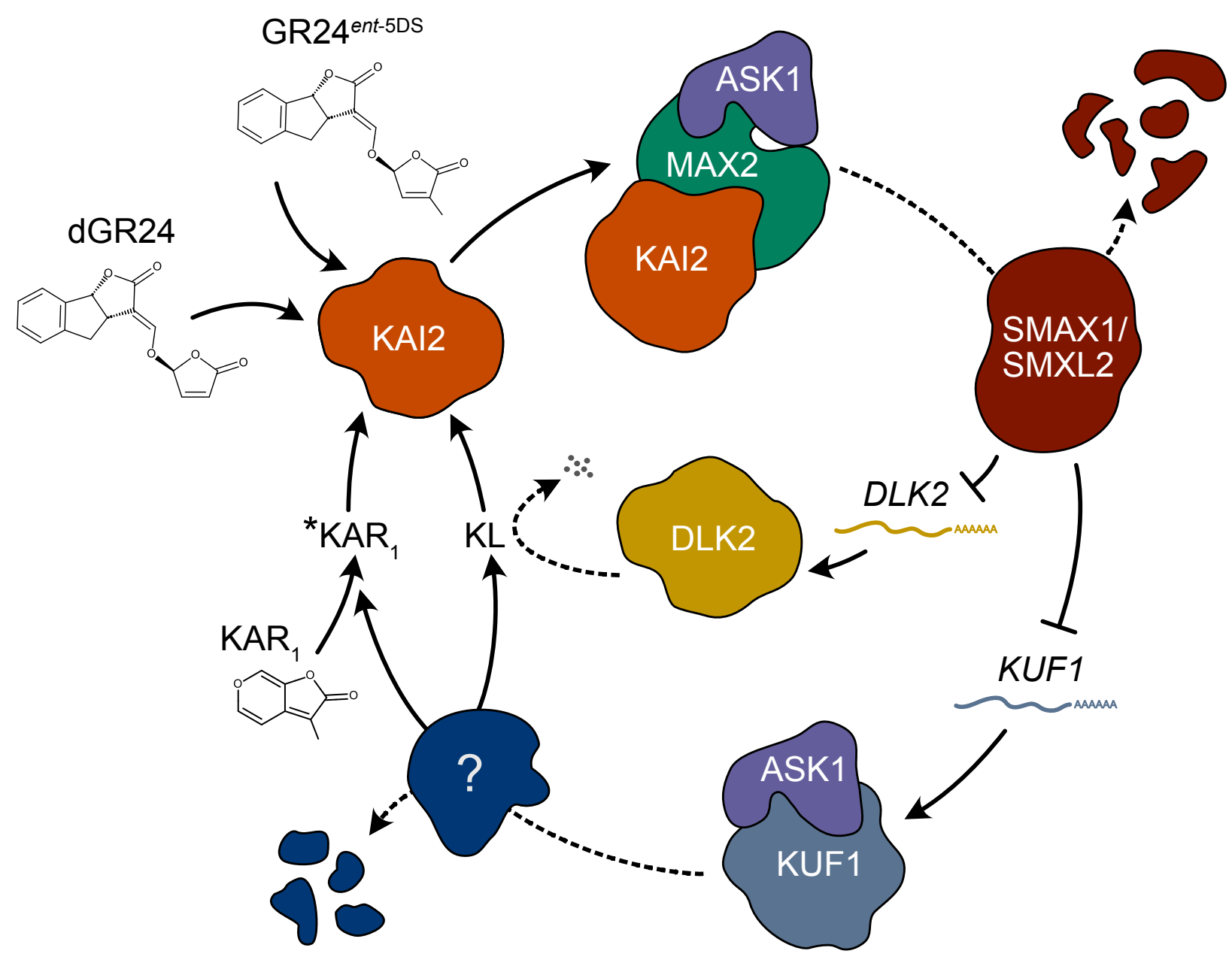

Figure S12. Model of KAR/KL signaling and feedback regulation by KUF1 and DLK2
KAI2 is activated by a hypothetical KAI2 ligand (KL), a hypothetical KAR metabolite, or synthetic molecules such as GR24$^{ent\text{-}5DS}$ or desmethyl-GR24 (dGR24). This causes KAI2 to interact with SCF^{MAX2} and SMAX1 or SMXL2. SMAX1/SMXL2 are polyubiquitinated by the SCF-type E3 ubiquitin ligase complex and degraded. Subsequent changes in gene expression include upregulation of the F-box protein-encoding gene *KUF1* and the *KAI2* homolog *DLK2*. SCF^{KUF1} negatively regulates KL/KAR signaling, putatively by reducing KL biosynthesis and KAR_1 metabolism into a bioactive KAI2 signal. KUF1 may target one or more protein substrates for polyubiquitination and degradation. DLK2 also negatively regulates KAR/KL signaling, putatively by hydrolyzing KL.

Table S1. RNA-seq statistics

		Input reads	Uniquely mapped reads
Col-0 mock	rep 1	31,499,832	28,452,789
	rep 2	46,491,702	40,299,314
	rep 3	45,526,773	41,227,951
	rep 4	47,476,465	43,408,623
Col-0 KAR2	rep 1	43,542,559	40,507,579
	rep 2	41,178,624	36,119,990
	rep 3	44,883,231	38,640,802
	rep 4	77,329,340	71,461,341
dlk2	rep 1	47,374,521	43,871,787
	rep 2	39,205,148	32,303,584
	rep 3	47,327,656	42,979,597
	rep 4	36,354,385	32,346,999
kai2	rep 1	53,207,010	48,516,944
	rep 2	53,287,317	44,348,299
	rep 3	54,996,361	50,662,553
	rep 4	54,847,439	49,009,606
kuf1	rep 1	44,708,678	39,921,078
	rep 2	60,049,185	44,112,991
	rep 3	53,424,485	48,046,562
	rep 4	54,233,785	43,616,040
kuf1 dlk2	rep 1	46,487,486	42,011,363
	rep 2	56,498,125	35,518,050
	rep 3	50,048,988	44,514,673
	rep 4	46,356,394	41,099,364
smax1 smxl2	rep 1	56,701,827	48,843,332
	rep 2	44,791,307	36,415,281
	rep 3	41,711,582	29,480,913
	rep 4	48,416,224	28,208,604

Table S2. Numbers of differentially expressed genes

		|FC| ≥ 1.5, adj p ≤ 0.05	|FC| ≥ 2, adj p ≤ 0.01
Col-0 KAR2	up	502	105
	down	637	261
dlk2	up	11	4
	down	6	6
kai2	up	604	251
	down	849	357
kuf1	up	218	54
	down	310	116
kuf1 dlk2	up	1010	349
	down	836	319
smax1 smxl2	up	1662	641
	down	1917	876

Table S3: Comparison of enzyme kinetic parameters towards the hydrolysis of Yoshimulactone Green (YLG) and desmethyl Yoshimulactone Green (dYLG). Michaelis-Menten parameters are shown with 95% confidence intervals in parentheses. Parameters were denoted ‘not defined’ (n.d.) where they were not possible to estimate, or where R^2 was below 0.5.

**Protein**	**YLG**			**dYLG**		
	V_{max}	K_m (µM)	R^2	V_{max}	K_m (µM)	R^2
AtKAI2	(n.d.)	(n.d.)	0.636	14.0 (12.9 – 15.0)	0.288 (0.151 – 0.465)	0.886
AtDLK2	(n.d.)	(n.d.)	0.165	4.17 (2.93 – 7.94)	16.8 (8.33 – 45.5)	0.904
BtDLK2	(n.d.)	(n.d.)	0.636	11.3 (8.55 – 17.3)	25.2 (15.8 – 46.5)	0.974
Btdlk2^{S102A}	(n.d.)	(n.d.)	0.150	(n.d.)	(n.d.)	0.0210
OsD14L2	1.40 (1.19 – 1.63)	0.851 (0.401 – 1.61)	0.740	19.0 (16.4 – 22.1)	1.69 (0.961 – 2.91)	0.832

Table S4. Primers used in this study

Primer Name	Primer Sequence (5' to 3')
**Genotyping**	
MW328_F	CTTACTTCTCTGCAGTCTACG
MW329_R	GATGTCATCTGTGGAAAATGC
LBa1	TGGTTCACGTAGTGGGCCATCG
max2-1-dCAPS-F	TGTCCGAATTTGGAAGAGATTAGG
max2-1-dCAPS-R	CAAGAAGAATCTTTCCCATAAACTCGAAT
d14-1-WiscLoxHS-LP	AAGAATATGGCAAGTGCAAC
d14-1-WiscLoxHS-RP	GATGATTCCGATCATAGCG
L4_WiscLoxHS	TGATCCATGTAGATTTCCCGGACATGAAG
htl-3-F	CACTTGGTTCCACATCTGGTC
htl-3-R	GAGATTTGAGTAACGATCGAAGTCG
kuf1-CR-F	CCTCTCATTCATTACACTTCACGC
kuf1-CR-R	CGACGGTTGATGAGAACTGAGAA
**Construct cloning**	
DLK2-3C-F	GAACCAATTCAGTCGACTATGGTGGTTAATCAGAAGATAT
DLK2-NLS/NES-R	GTCTCGGGCTTCATCTTCTT
mTurquoise2-3C-F	GAACCAATTCAGTCGACTATGGTTTCAAAGGGAGAA
mTurquoise2-3C-R	CGAGTGCGGCCGCGAATTTTATTTATAAAGCTCATCCATAC
DLK2-cLUC-F	TCGTACGCGTCCCGGGGCATGGTGGTTAATCAGAAGATAT
DLK2-cLUC-R	CGAACGAAAGCTCTGCAGTTAAAACTCAAGGAGGCG
DLK2-nLUC-F	AACACGGGGGACGAGCTCATGGTGGTTAATCAGAAGATAT
DLK2-nLUC-R	GGACGCGTACGAGATCTGAAACTCAAGGAGGCGC
pSUMO F	CGACGGTATTAGAATTCAAGC
pSUMO R	GCTAGTTATTGCTCAGCGGT
BtDLK2 S102A F	GTTTGTTGGTCACGCCATGTCAGG
BtDLK2 S102A R	ACGACCGGACCCAGTTTCAGCTC

Dataset S1. Differentially expressed genes identified in transcriptome analysis (separate file). Tables show differentially expressed genes defined as fold-change ≥ 1.5 or ≤ -1.5 relative to control (Col-0, mock-treated) AND adjusted p-value (FDR) < 0.05. Differential expression analysis was performed with DEseq2 in RStudio after read-mapping with STAR. AGI, Arabidopsis Gene Identifier. baseMean refers to normalized read counts, not FPKM or RPKM or TPM. Genes have not been filtered for low baseMean read counts, which may affect fold-changes. Gene annotations are from http://bar.utoronto.ca/ntools/cgi-bin/ntools_agi_converter.cgi

Dataset S2. Sequences of synthesized genes
